# Minor Effects of 11 *Dof* Family Genes Contribute to the Missing Heritability of Heading Date in Rice (*Oryza sativa* L.)

**DOI:** 10.3389/fpls.2019.01739

**Published:** 2020-01-24

**Authors:** Yong Huang, Zhongmin Han, Niannian Cheng, Meifang Luo, Xufeng Bai, Yongzhong Xing

**Affiliations:** National Key Laboratory of Crop Genetic Improvement, Huazhong Agricultural University, Wuhan, China

**Keywords:** rice, *Dof* family, heading date, haplotype-based association, knockout mutants, missing heritability

## Abstract

DNA binding with one finger (Dof) proteins are plant-specific transcription factors with important and diverse functions in seed germination, flowering time, and biotic and abiotic stresses. In this study, haplotype-based association analysis was conducted between heading date and 30 *Dof* family genes in a worldwide germplasm collection. Of these, 22 *Dof* genes were associated with heading date. Multiple comparisons among haplotypes revealed their diverse functions in promoting and suppressing heading date under short-day (SD) and long-day (LD) conditions. They cumulatively made a considerable contribution to the missing heritability of heading date. A set of knockout mutants of 30 *Dof* genes generated by CRISPR/Cas9-mediated genome editing technology showed that 11** and 9 *Dof* genes regulated heading date under LD and SD, respectively. Phenotype measurement of mutants showed that these 11 and 9 *Dof* genes slightly regulated heading with effects of 2–5 days under LD and SD, respectively. Both mutant and natural variation assays indicated functional redundancy in regulating heading date among *Dof* family genes. Nucleotide diversity analysis suggested that most *Dof* genes have been subjected to selection during domestication and improvement. Beyond heading date, this set of mutants is also a good resource for evaluating the function of *Dof* genes in regulating stress tolerance and seed germination.

## Introduction

Functional characterization of gene families in crops is important. Genes in the same family frequently have conserved functions (Jain et al., 2006; Li et al., 2016; Dong et al., 2018). However, in some cases, genes within a family have very distinct functions (Péret et al., 2012; Park et al., 2017; Zhang et al., 2017). For example, the *AUXIN* (*AUX*)/*LIKE AUX* (*LAX*) genes in *Arabidopsis* encode a family of auxin influx transporters comprising four highly conserved genes, *AUX1*, *LAX1*, *LAX2*, and *LAX3*. *AUX1*, *LAX1*, and *LAX3* regulate distinct auxin-dependent developmental processes, but *LAX2* regulates vascular patterning in cotyledons (Péret et al., 2012).

Functional characterization of gene families is often made by overexpression, knockout (gene editing), or knockdown (RNA interference). Overexpression sometimes causes ectopic expression and does not necessarily reflect the native function of target genes. Knocking out and knocking down genes are better ways to identify gene function because these methods precisely target the gene *in situ*, which does not cause genetic noise. However, the effect of manipulating single genes is reduced if functional redundancy exists within the gene family. Currently, the clustered regularly interspaced short palindromic repeats (CRISPR) system is a powerful tool to produce mutants in target genes (Cong et al., 2013; Hwang et al., 2013; Shan et al., 2013; Yin et al., 2017; Zhou et al., 2018). CRISPR technology relies on Cas9 and single guide RNA (sgRNA) to achieve precise cutting. CRISPR/Cas9 systems have been applied to multiplexed genome editing (Ma et al., 2015; Zhang et al., 2016), which will facilitate functional analysis of gene families. Transgene-free plants can be achieved through backcrossing and used directly in breeding. For example, the non-transgenic, low-gluten wheat lines were obtained through modification of the α-gliadin family genes using CRISPR/Cas9 technology and serve as source material to introgress this trait into elite wheat varieties (Sanchez-Leon et al., 2018).

Next-generation sequencing technology has developed rapidly and has greatly facilitated genome-wide association studies (GWAS) (Huang et al., 2010; Huang et al., 2017), which have been widely used to identify candidate genes associated with various complex traits in several species including humans, animals, and plants (Wellcome Trust Case Control C, 2007; Moreira et al., 2018; Su et al., 2018). Candidate gene-based association analysis is an option to identify potential functions of related genes. Moreover, association mapping at the haplotype level is a very effective method to establish the relationships between genes and traits (Han et al., 2016; Chen et al., 2018; Lu et al., 2018). Haplotype development is easy at the single gene level, which is helpful to identify the association between related genes and traits at the haplotype level.

DNA binding with one finger (Dof) proteins are plant-specific transcription factors (Yamagishi et al., 2002; Yamaguchi et al., 2004). Since the first *Dof* gene, *MNB1*, was identified in maize (Yanagisawa and Izui, 1993), an increasing number of *Dof* genes have been identified in various plant species. Recent studies have revealed the role of the *Dof* family genes in multiple plant developmental processes. In tomato, *TDDF1* (*TOMATO DOF DAILY FLUCTUATIONS 1*) is involved in circadian regulation and stress resistance (Ewas et al., 2017). In maize, *ZmDof3* regulates starch accumulation and aleurone development in maize endosperm (Qi et al., 2017). In rice, a total of 30 *Dof* genes have been predicted through genome analysis (Lijavetzky et al., 2003). All have a highly conserved DNA-binding domain (Dof domain) of 50 amino acid residues including a C2C2-type zinc finger motif (Huang et al., 2018). *RDD1*/*OsDof2* overexpression promotes flowering in addition to regulating nutrient ion uptake and accumulation (Iwamoto et al., 2009; Iwamoto and Tagiri, 2016). *OsDof4* has distinct flowering effects under long-day and short-day conditions (Wu et al., 2017). *OsDof12* participates in the regulation of flowering time and plant architecture (Li et al., 2009; Wu et al., 2015). These studies indicate that *Dof* family genes in rice might have important functions in flowering. Therefore, we hypothesized that some other *Dof* family genes are probably related to flowering. To test this hypothesis, association analysis between heading date and haplotypes of *Dof* family genes were conducted in this study. On the one hand, candidate gene-based association analysis of *Dof* family genes with heading date was examined at the haplotype level in a worldwide germplasm collection of 529 rice accessions. On the other hand, the CRISPR/Cas9 gene editing technology was utilized to test which *Dof* family genes really function in the regulation of heading date. Our results showed that 11 and 9 *Dof* genes regulated heading date under long day (LD) and short day (SD), respectively. Nucleotide diversity analysis showed that most *Dof* genes regulating heading date have been subjected to selection during domestication and genetic improvement.

## Materials and Methods

### Plant Materials and Field Experiments

Zhonghua 11 (ZH11; *Oryza sativa japonica*) was used as the recipient for transformation. A total of 529 rice accessions from around the world, composed of a core Chinese collection of 202 cultivars and a core world collection of 327 cultivars, were grown in Wuhan (114°21′E, 30°28′N; the average day length is more than 13.5 h from the middle of May to the beginning of August; 23–31°C) in the summer of 2013 and Hainan (110°01′E, 18°30′N; the average day length was less than 12.5 h from December to the middle of March; 20–28°C) in the winter of 2013 (https://www.timeanddate.com, https://www.climatestotravel.com). The details of field management and measurement of heading date are described in a previous study (Han et al., 2016). The 529 accessions were genotyped *via* sequencing and classified into four subpopulations: *indica*, *aus*, *japonica*, and Admixture (Chen et al., 2014). Single nucleotide polymorphism (SNP) information about the 529 cultivars is available on RiceVarMap v1.0 (http://ricevarmap.ncpgr.cn/v1/), which is a comprehensive database of rice genomic variations. All transgenic plants were grown in the field in Wuhan (LD) in summer 2018 and Hainan (SD) in spring 2019. Heading date was individually scored as the number of days from sowing to the emergence of the first panicle on the plant. Plant height was measured from the surface to the top of the main panicle.

### Haplotype Construction and Haplotype-Based Association Analysis

To obtain sequencing data for all *Dof* genes in rice, the conserved Dof domain sequence of the known protein SP3 was used to search the Rice Genome Annotation Project (http://rice.plantbiology.msu.edu) and NCBI (https://www.ncbi.nlm.nih.gov/) (Huang et al., 2018). A total of 30 *Dof* genes were identified.

For development of haplotypes, first, SNPs in *Dof* genes were extracted from the sequences containing a 2-kb promoter region and the gene body region. Then, PHASE software, which can estimate missing and heterozygous genotypes, was used to construct haplotypes (Stephens et al., 2001). Finally, the haplotypes with an allele frequency ≥0.01 (five accessions) were included for association analysis. Analysis of variance (ANOVA) was used to identify the association between haplotype and heading date. Duncan's test was employed to perform multiple comparisons between all possible haplotype pairs. The phenotypic variation explained by these 22 *Dof* genes was calculated by ANOVA with a linear model including all these genes.

### Nucleotide Diversity Analyses

DNA sequences of each *Dof* family gene in 1,612 cultivar accessions and 446 *Oryza rufipogon* accessions were obtained from the ECOGEMS database (http://ecogems.ncpgr.cn). Each sequence contained a 2-kb promoter region and a gene body region. The details of these accessions and their sequencing data have been previously reported (Huang et al., 2012a). The nucleotide diversity (*π*), Tajima's *D*, and Fu and Li's *D* statistics were calculated both in cultivars and wild rice (*O. rufipogon*) using the DnaSP 6.0 program (Rozas et al., 2017).

### Vector Construction and Transformation

To generate knockout mutants of *Dof* family genes by CRISPR/Cas9, 19- and 20-bp fragments in the exon regions of *Dof* genes were chosen as the candidate targets according to the design principles of the target sequences in the CRISPR/Cas9 system (**Supplementary Table S1**). At least one target was in the exon regions of each *Dof* gene (**Supplementary Figure S1**). The two target fragments were introduced into two sgRNA expression cassettes by dual-nested PCR, driven by the OsU6a and OsU6b promoters, respectively. Then, the multiple sgRNA expression cassettes were ligated to the CRISPR/Cas9 binary vector (pYLCRISPR/Cas9Pubi-H) based on Golden Gate cloning (Ma et al., 2015). Finally, 30 recombinant CRISPR/Cas9 constructs were introduced into *Agrobacterium tumefaciens* strain EHA105 and separately transferred into ZH11 by *Agrobacterium*-mediated transformation (Lin and Zhang, 2005).

### Mutation Detection and Transgene-Free Line Screening

T_0_ transgenic plants were used to detect mutations. Genomic DNA was extracted from the leaves of T_0_ plants using the cetyltrimethyl ammonium bromide (CTAB) method. Primer pairs flanking the designated target sites were used to amplify the potentially mutated fragments. PCR products were sequenced to detect mutations. Homozygous mutations were identified using the Sequencher 5.1 Demo software and Degenerate Sequence Decoding method (DSDecodeM; Liu et al., 2015). In our study, the homozygous transgenic knockout lines were transgene-free (or transgene-clean) mutant lines, which were obtained by screening the recombinants between Cas9 and the targeted mutant gene in the selfing progeny. Thus, the phenotypic evaluation was performed between transgene-clean mutant lines and wild type, not the negative plants. The seeds were harvested from each homozygous T_0_ plant, and the transgene-free plants were identified from T_1_ homozygous families by agarose gel electrophoresis to separate PCR products with Cas9-specific primers. Cas9-negative plants were transgene-free. CRISPR/Cas9 plasmid DNA was used as a positive control, and ZH11 DNA and H_2_O were used as negative controls.

## Results

### Gene Structure and Haplotype Analysis of *Dof* Family Genes

The 30 *Dof* genes were not evenly distributed in the genome. Chromosomes 1 and 3 each had the largest number of genes (six), and no *Dof* genes were located on chromosome 11 (**Table 1**). In general, *Dof* family genes had relatively simple structures. Of all *Dof* genes, 17, 12, and 1 had no intron, one intron, and two introns, respectively (**Supplementary Figure S1**). The number of SNPs in the 2-kb promoter and entire genomic sequence of *Dof* family genes ranged from 14 (*OsDof14*) to 113 (*OsDof10*; **Table 1**). Based on these SNPs, we constructed the haplotypes of all 30 genes in the germplasm collection of 529 accessions. In the full population, the number of haplotypes ranged from 4 to 20, among which *OsDof10* had the largest number (twenty) of haplotypes, whereas *OsDof18* had the lowest number (four) of haplotypes. Additionally, with the exception of *OsDof4*, *OsDof21*, *OsDof29*, and *OsDof30*, all *Dof* genes had more haplotypes in the *indica* subpopulation than the *japonica* subpopulation (**Table 1**).

**Table 1:** Genome positions of *Dof* family genes and their numbers of SNPs and haplotypes in 529 accessions.

Gene name	Gene ID	Chr.	Genome position	N-SNP	N-Hap (F, I, J)
*OsDof1*	LOC_Os01g64590	1	37,473,368–37,472,376	24	(8, 6, 5)
*OsDof2*	LOC_Os01g15900	1	8,952,712–8,949,249	70	(13, 10, 6)
*OsDof3*	LOC_Os01g09720	1	5,014,609–5,015,256	33	(10, 7, 4)
*OsDof4*	LOC_Os01g17000	1	9,733,832–9,738,226	84	(10, 5, 7)
*OsDof5*	LOC_Os01g48290	1	27,676,501–27,677,028	17	(6, 4, 4)
*OsDof6*	LOC_Os01g55340	1	31,851,619–31,850,537	39	(12, 7, 6)
*OsDof7*	LOC_Os02g47810	2	29,241,762–29,238,064	37	(11, 10, 3)
*OsDof8*	LOC_Os02g49440	2	30,208,306–30,206,964	25	(7, 4, 4)
*OsDof9*	LOC_Os02g45200	2	27,438,687–27,435,303	41	(7, 4, 4)
*OsDof10*	LOC_Os02g15350	2	8,593,925–8,590,287	113	(20, 15, 12)
*OsDof11*	LOC_Os03g38870	3	21,599,677–21,600,792	50	(10, 6, 4)
*OsDof12*	LOC_Os03g07360	3	3,738,779–3,741,715	37	(8, 5, 2)
*OsDof13*	LOC_Os03g42200	3	23,475,827–23,472,278	83	(10, 8, 3)
*OsDof14*	LOC_Os03g16850	3	9,360,935–9,359,466	14	(7, 4, 4)
*OsDof15*	LOC_Os03g55610	3	31,663,961–31,662,275	27	(8, 8, 2)
*OsDof16*	LOC_Os03g60630	3	34,455,535–34,456,959	28	(9, 6, 4)
*OsDof17*	LOC_Os04g58190	4	34,656,922–34,655,221	78	(10, 10, 3)
*OsDof18*	LOC_Os04g47990	4	28,537,554–28,534,300	32	(4, 3, 2)
*OsDof19*	LOC_Os05g02150	5	660,255–658,106	78	(11, 8, 5)
*OsDof20*	LOC_Os06g17410	6	10,101,312–10,102,739	108	(13, 8, 8)
*OsDof21*	LOC_Os07g13260	7	7,605,698–7,604,087	42	(13, 6, 9)
*OsDof22*	LOC_Os07g32510	7	19,362,157–19,364,739	28	(10, 7, 6)
*OsDof23*	LOC_Os07g48570	7	29,081,179–29,077,196	57	(13, 11, 7)
*OsDof24*	LOC_Os08g38220	8	24,232,676–24,233,972	30	(5, 3, 2)
*OsDof25*	LOC_Os09g29960	9	18,234,924–18,235,879	58	(10, 8, 6)
*OsDof26*	LOC_Os10g26620	10	13,885,224–13,882,332	48	(10, 8, 2)
*OsDof27*	LOC_Os10g35300	10	18,875,014–18,873,473	97	(8, 7, 6)
*OsDof28*	LOC_Os12g38200	12	23,458,337–23,460,708	74	(10, 7, 4)
*OsDof29*	LOC_Os05g36900	5	21,555,554–21,556,537	56	(11, 4, 7)
*OsDof30*	LOC_Os12g39990	12	24,724,587–24,723,790	39	(7, 4, 5)

N-SNP is the number of SNPs detected in the 2-kb promoter and entire genomic sequence in the germplasm collection. N-Hap (F, I, J) is the number of haplotypes in the full population, indica, and japonica subpopulations, respectively.

### Haplotype-Based Association Analysis of *Dof* Genes With Heading Date

Heading date had a high heritability of 0.75 across both conditions. However, SNP-based GWAS only identified a few loci that were significantly associated with heading date, and they only explained a small fraction of heading date variance (Han et al., 2016). *Dof* family genes were not significantly associated with heading date in GWAS (**Supplementary Figure S2**). Candidate gene-based association analysis for heading date showed that more *Dof* genes could be associated with heading date under LD and SD conditions at the haplotype level (**Table 2**). Under LD, 17 genes (*OsDof1*, *3*, *6*, *7*, *8*, *10*, *11*, *12*, *13*, *16*, *20*, *21*, *22*, *23*, *25*, *29*, and *30*), 10 genes (*OsDof1*, *3*, *6*, *7*, *8*, *11*, *13*, *16*, *20*, and *21*) and 7 genes (*OsDof7*, *10*, *11*, *12*, *20*, *23*, and *25*) were significantly associated with heading date in the full population, both the full population and the *indica* subpopulation, and both the full population and the *japonica* subpopulation, respectively. Three genes (*OsDof7*, *11*, and *20*) were commonly identified in the full population, the *indica* subpopulation, and the *japonica* subpopulation (**Table 2**). Under SD, 21 genes (*OsDof2*, *3*, *6*, *7*, *8*, *10*, *11*, *12*, *13*, *14*, *16*, *19*, *20*, *21*, *22*, *23*, *25*, *26*, *27*, *29*, and *30*), 7 genes (*OsDof2*, *6*, *10*, *11*, *14*, *16*, and *20*), and 14 genes (*OsDof2*, *6*, *7*, *10*, *11*, *19*, *20*, *21*, *22*, *23*, *25*, *27*, *29*, and *30*) were significantly associated with heading date in the full population, both the full population and the *indica* subpopulation, and both the full population and the *japonica* subpopulation, respectively. Five genes (*OsDof2*, *6*, *10*, *11*, and *20*) were commonly associated with heading date in the full population, *indica* subpopulation, and *japonica* subpopulation (**Table 2**). Moreover, 16 genes (*OsDof3*, *6*, *7*, *8*, *10*, *11*, *12*, *13*, *16*, *20*, *21*, *22*, *23*, *25*, *29*, and *30*) were commonly associated with heading date in the full population under both LD and SD. Two genes (*OsDof11* and *20*) were commonly detected in the full population and two subpopulations under both conditions (**Table 2**). These 22 *Dof* genes cumulatively explained about 18.4% of the variation in heading date in the whole collection.

**Table 2:** Haplotype-based association analyses of *Dof* family genes for heading date in different environments.

Gene	Population	*P*(2013WH)	*P*(2013HN)	Gene	Population	*P*(2013WH)	*P*(2013HN)
*OsDof1*	All	9.2E−05		*OsDof14*	All		1.54E−08
	*Ind*	1.5E−06			*Ind*		1.0E−07
*OsDof2*	All		2.4E−17	*OsDof16*	All	3.9E−09	2.0E−12
	*Ind*		5.2E−05		*Ind*	1.7E−06	4.3E−05
	*Jap*		6.9E−10	*OsDof19*	All		5.5E−16
*OsDof3*	All	9.7E−06	1.8E−05		*Jap*		1.2E−06
	*Ind*	1.6E−06		*OsDof20*	All	2.6E−16	4.3E−22
*OsDof6*	All	2.1E−16	2.0E−24		*Ind*	6.3E−12	7.4E−11
	*Ind*	1.3E−18	3.2E−05		*Jap*	5.2E−05	1.9E−05
	*Jap*		1.7E−16	*OsDof21*	All	1.1E−06	4.3E−26
*OsDof7*	All	9.8E−17	7.1E−14		*Ind*	8.6E−07	
	*Ind*	3.4E−09			*Jap*		2.9E−13
	*Jap*	1.1E−07	2.8E−12	*OsDof22*	All	2.5E−07	2.1E−21
*OsDof8*	All	4.9E−05	3.3E−08		*Jap*		2.9E−09
	*Ind*	6.3E−07		*OsDof23*	All	1.3E−06	1.3E−24
*OsDof10*	All	5.3E−12	3.1E−27		*Jap*	5.6E−07	1.5E−17
	*Ind*		4.8E−08	*OsDof25*	All	7.4E−12	4.9E−30
	*Jap*	7.0E−06	6.4E−11		*Jap*	7.6E−11	1.5E−20
*OsDof11*	All	2.3E−11	2.6E−19	*OsDof26*	All		6.8E−06
	*Ind*	6.4E−07	5.7E−07	*OsDof27*	All		1.5E−21
	*Jap*	4.4E−06	2.3E−05		*Jap*		9.1E−13
*OsDof12*	All	1.2E−10	1.5E−07	*OsDof29*	All	9.4E−06	9.1E−18
	*Jap*	7.4E−08			*Jap*		1.1E−12
*OsDof13*	All	5.9E−07	8.8E−10	*OsDof30*	All	6.2E−05	7.5E−23
	*Ind*	1.6E−06			*Jap*		2.3E−21

WH, Wuhan; HN,Hainan; Ind, indica; Jap, japonica. P is the P value from the ANOVA.

### Comparison of Haplotype Effects on Heading Date

Multiple comparisons of haplotypes were made for heading date-associated genes. Under LD, significant differences in heading date were detected among major haplotypes of 10 genes (*OsDof1*, *6*, *7*, *10*, *11*, *12*, *13*, *16*, *20*, and *21*) in both *indica* and *japonica* accessions (**Supplementary Table S2**). Under SD, a similar result was also observed in these 10 genes except for *OsDof1* and *OsDof12* (**Supplementary Table S2**).

**Figure 1:**
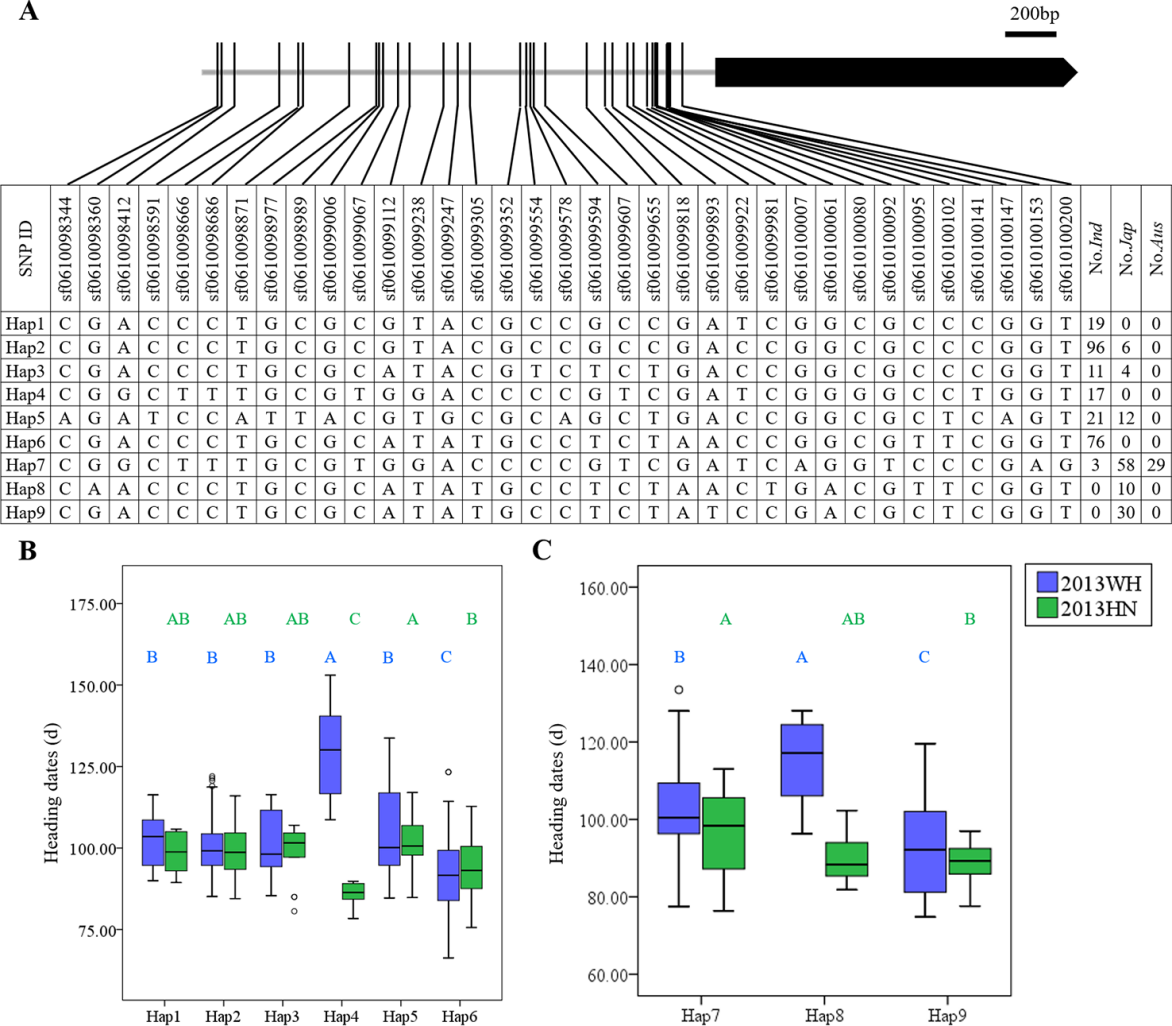
Haplotype analysis of *OsDof20*. **(A)** Major haplotypes (each haplotype contains more than 10 accessions) of *OsDof20* in the full population according to SNPs data from RiceVarMap version 1. The region contains 2 kb upstream and coding region. **(B)** Comparison of haplotype effects in heading dates among Hap1–Hap6 in *indica* rice using a Duncan's test (*P* < 0.01), respectively. **(C)** Comparison of heading dates among Hap7–Hap9 in *japonica* rice using a Duncan's test (*P* < 0.01), respectively.

For example, in *OsDof20*, nine major haplotypes were obtained, and most *indica* accessions were classified into Hap1, Hap2, Hap3, Hap4, Hap5, and Hap6. The majority of the *aus* and *japonica* accessions carried Hap7, Hap8, and Hap9 (**Figure 1A**). In *indica* rice, Hap4 produced significantly delayed heading (127.7 ± 7.2 days) under LD and promoted heading (85.9 ± 3.7 days) under SD compared to other haplotypes (**Figure 1B**). In *japonica* rice, Hap8 significantly delayed heading date (114.9 ± 11.4 days) compared to Hap9 (93.4 ± 13.6 days) and Hap7 (103.1 ± 11.3 days) under LD. Under SD, Hap9 (88.6 ± 5.3 days) significantly promoted heading date compared to Hap7 (96.1 ± 10.7 days) (**Figure 1C**). Although no SNPs causing amino acid changes were detected in the *OsDof20* coding region, some SNPs (sf0610098412, sf0610098666, sf0610099067, sf0610099607, sf0610100080, and sf0610100141) in the promoter region were associated with heading date (**Figure 1A**). Therefore, the genetic variation in the promoter regions of *OsDof20* may lead to a significant difference in heading date.

**Figure 2:**
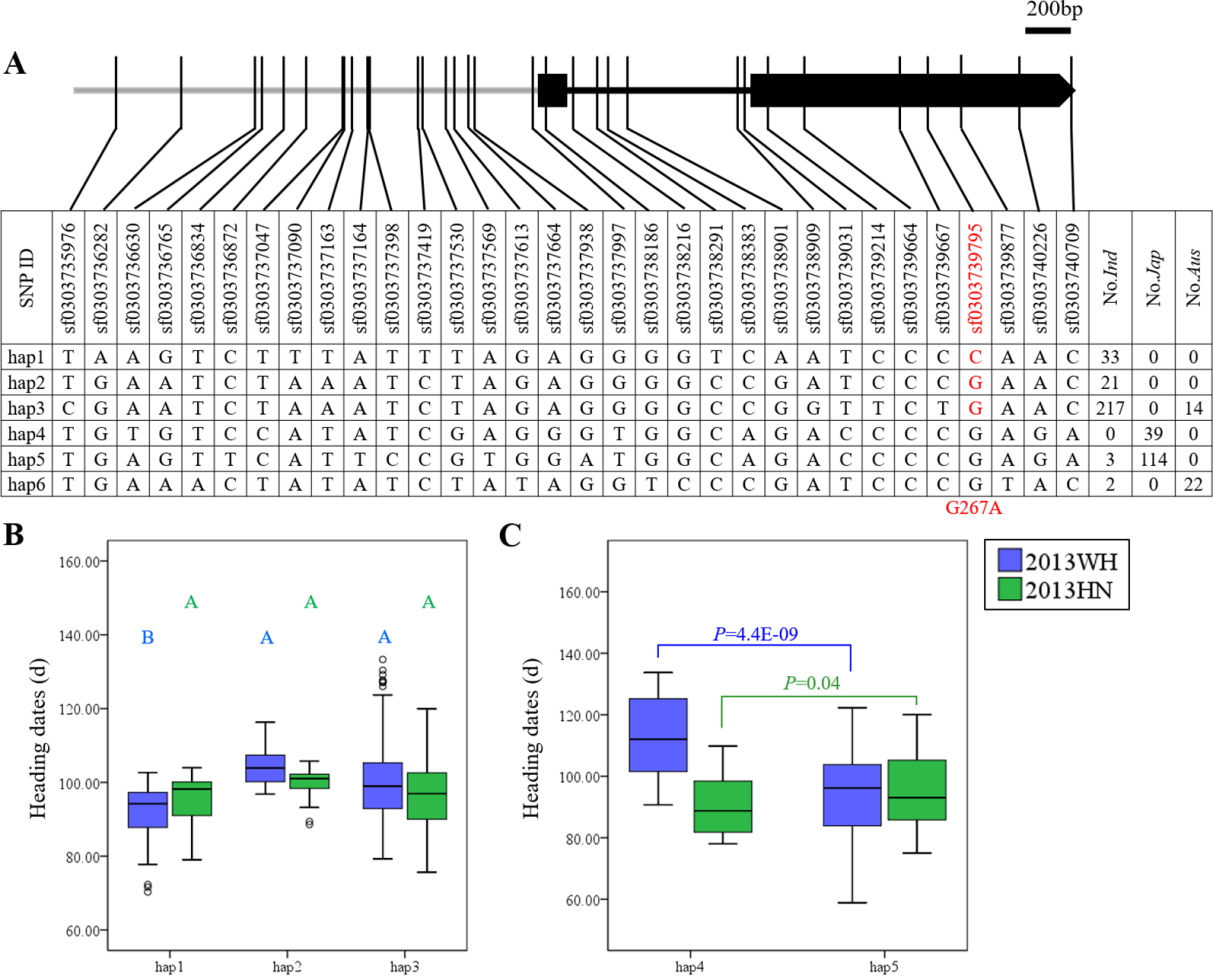
Haplotype analysis of *OsDof12*. **(A)** Major haplotypes (each haplotype contains more than 10 accessions) of *OsDof12* in the full population according to SNP data from RiceVarMap version 1. The region contains 2 kb upstream and coding region. The SNP in *red* is non-synonymous. **(B)** Comparison of haplotype effects in heading dates among Hap1–Hap3 in *indica* rice using a Duncan's test (*P* < 0.01), respectively. **(C)** Comparison of heading dates between Hap4 and Hap5 in *japonica* rice by Student's *t* test.

*OsDof12* had six major haplotypes in the germplasm collection. Hap1, Hap2, and Hap3 were mainly harbored by the *indica* subpopulation, Hap4 and Hap5 were primarily harbored by the *japonica* subpopulation, and most *aus* accessions belonged to Hap6 (**Figure 2A**). Under LD, Hap2 (104.4 ± 5.9 days) and Hap3 (101.0 ± 10.7 days) significantly delayed heading date compared to Hap1 (91.1 ± 9.1 days) within *indica* rice (**Figure 2B**), and Hap4 (114.8 ± 10.9 days) significantly delayed heading date compared to Hap5 (94.2 ± 13.2 days) in *japonica* rice (**Figure 2C**). These functionally diverse haplotypes provide us with an opportunity to improve rice heading date for specific ecotypes. However, under SD, there were no significant differences in heading date among major haplotypes in either *indica* or *japonica* rice (**Figures 2B, C**). Interestingly, only one SNP (sf0303739795) causing a non-synonymous mutation (G267A) was detected in the coding region of *OsDof12*, and most *aus*, *indica*, and *japonica* accessions carried Gly at site 267 (Hap2–Hap6). Only a small minority of *indica* accessions carried Hap1 with Ala at site 267 (**Figure 2A**). Within *indica* rice, Hap2 with G267 and Hap3 with G267 significantly delayed heading date compared to Hap1 with A267 in LD (**Figure 2B**). Therefore, G267A might be the causal mutation for the variation of heading date in *indica* rice. Accordingly, *OsDof1* had significant effects on heading date in both *indica* and in *japonica* rice under LD, whereas no significant effects were observed under SD (**Supplementary Table S2**).

**Figure 3:**
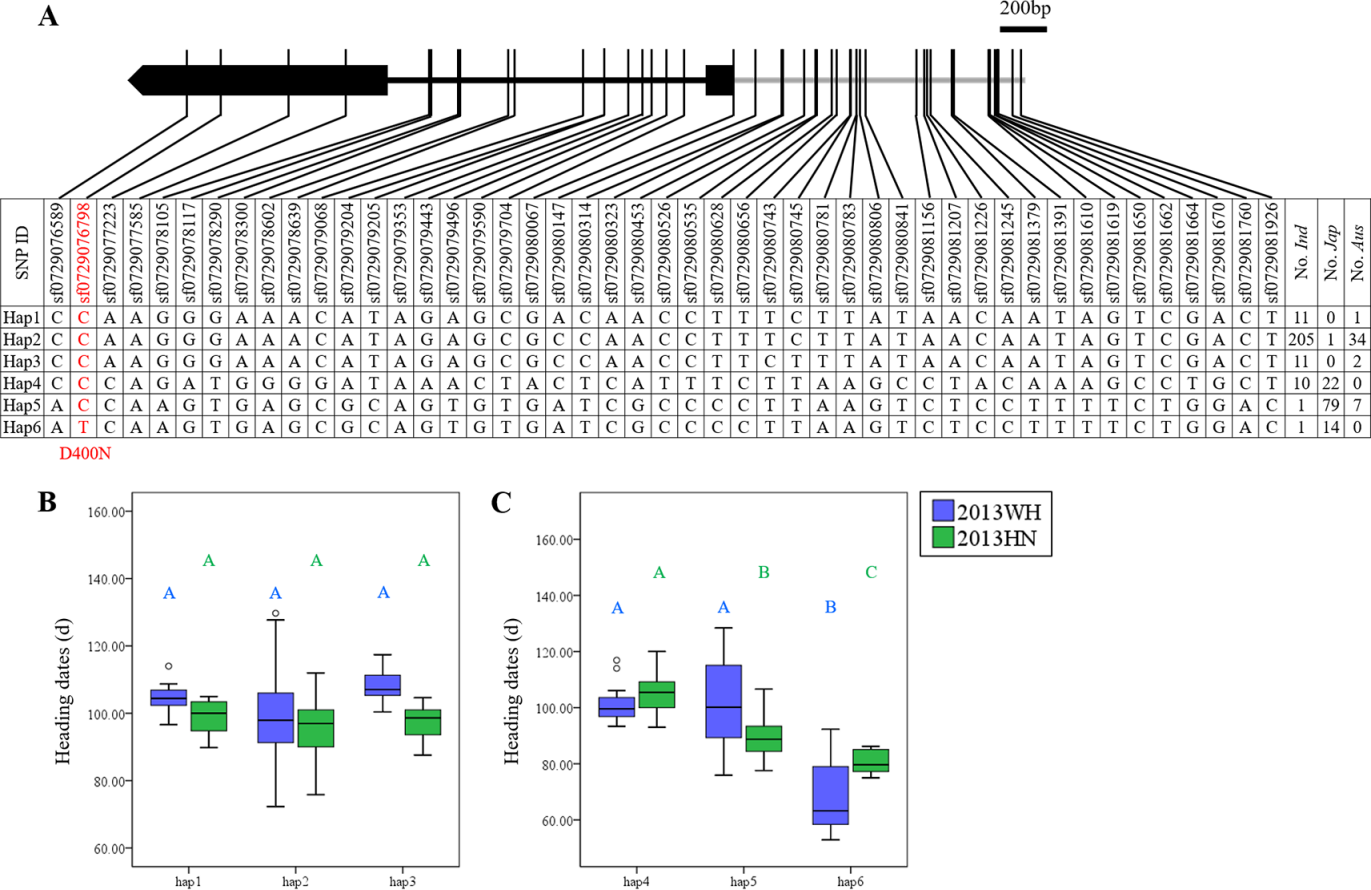
Haplotype analysis of *OsDof23*. **(A)** Major haplotypes (each haplotype contains more than 10 accessions) of *OsDof23* in the full population according to SNP data from RiceVarMap version 1. The region contains 2 kb upstream and coding region. The SNP in *red* is non-synonymous. **(B)** Comparison of haplotype effects in heading dates among Hap1–Hap3 in *indica* rice using a Duncan's test (*P* < 0.01), respectively. **(C)** Comparison of heading dates among Hap4–Hap6 in *japonica* rice using a Duncan's test (*P* < 0.01), respectively.

In addition, five genes (*OsDof2*, *23*, *25*, *29*, and *30*) had significant effects in *japonica* accessions and not in *indica* rice under SD (**Supplementary Table S2**). These five genes, except *OsDof2*, also had similar effects under LD (**Supplementary Table S2**). In the case of *OsDof23*, six major haplotypes were obtained. Hap1, Hap2, and Hap3 were mainly harbored by the *indica* subpopulation, whereas Hap4, Hap5, and Hap6 were primarily harbored by the *japonica* subpopulation (**Figure 3A**). Two SNPs (sf0729076798 and sf0729080067) in the coding region caused amino acid changes. The SNP at site sf0729076798 caused a non-synonymous mutation (D400N). Most *aus*, *indica*, and *japonica* accessions were divided into Hap1–Hap5 and shared an Asp at site 400. A small proportion of *japonica* accessions possessed Hap6 with Asn at site 400 (**Figure 3A**). No significant effects were detected in *indica* rice in either LD or SD (**Figure 3B**), whereas the effects were significant in *japonica* rice (**Figure 3C**). Hap6 with N400 (69.2 ± 9.9 days under LD and 81.6 ± 4.3 days under SD) significantly promoted heading date compared to Hap4 with D400 (100.4 ± 5.4 days under LD and 105.5 ± 7.2 days under SD) and Hap5 with D400 (104.8 ± 13.5 days under LD and 89.9 ± 7.7 days under SD; **Figure 3C**). Therefore, sf0729076798 might be a functional nucleotide polymorphism site in *japonica* rice.

### Mutagenesis of *Dof* Genes by CRISPR/Cas9

Multiple positive plants for each gene were harvested from T_0_ transgenic plants of all *Dof* family genes generated by the CRISPR/Cas9 system (**Supplementary Table S3**). Transgene-free homozygous knockout lines with various mutations in 30 *Dof* genes were identified by sequencing. The mutations included small fragment insertions (1 bp), deletions (1–17 bp), and large fragment deletions (111–2,371 bp; **Supplementary Figure S3**). Because the two target sites were designed in the exon region (at least one target fell in the coding region) of *Dof* genes, each mutation occurred in the coding region and led to a frameshift or truncation mutation in the protein that may completely deactivate the protein function.

### Identification of *Dof* Family Genes Regulating Heading Date Using Mutants

**Figure 4:**
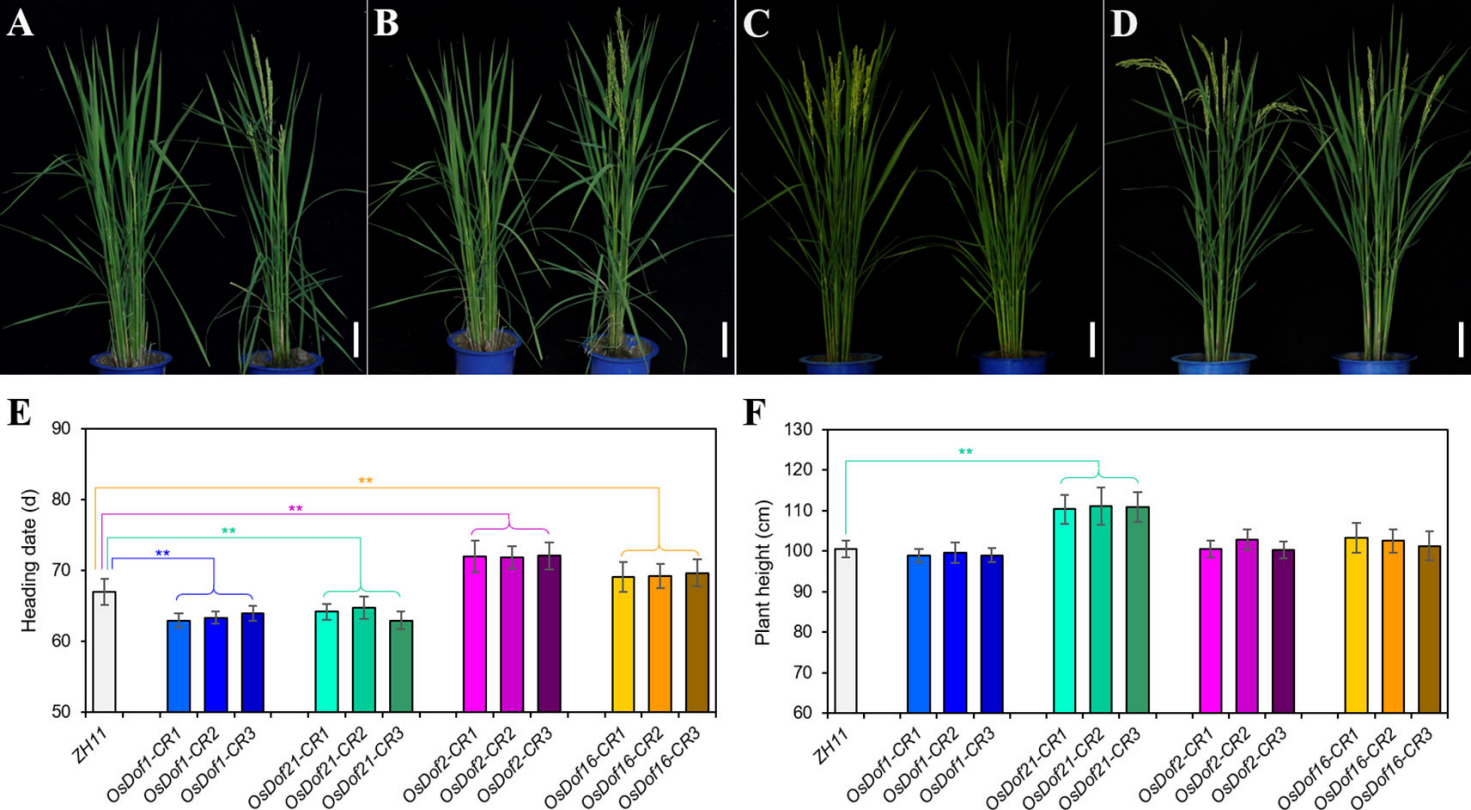
Performance of *Dof* gene mutants generated by utilizing CRISPR/Cas9 technology. **(A–D)** Plant appearance of *OsDof1*-CRISPR (*OsDof1*-CR) **(A)**, *OsDof21*-CRISPR (*OsDof21*-CR) **(B)**, *OsDof2*-CRISPR (*OsDof2*-CR) **(C)**, and *OsDof16*-CRISPR (*OsDof16*-CR) **(D)** transgenic plants [all homozygous mutants in the T_1_ generation (*right*) and ZH11 wild type (*left*)], respectively. **(E, F)** Comparison of heading dates **(E)** and plant height **(F)** between the mutants (*OsDof1*-CR, *OsDof21*-CR, *OsDof2*-CR, and *OsDof16*-CR) and ZH11 wild type, respectively. **P < 0.01 [*n* = 20 plants for **(E, F)**; Student's *t* test]. *Scale bars* = 10 cm in **(A–D)**.

At least three independent mutant lines for each *Dof* gene were analyzed for heading date. Compared with the wild-type ZH11, the CRISPR/Cas9 mutated plants of four *Dof* family genes (*OsDof1*, *11*, *21*, and *29*) promoted heading by approximately 3–4 days under LD (**Figures 4A, B, E**, **Table 3**, and **Supplementary Figure S4**). Under SD, a similar result was also observed in these four genes except for *OsDof11* and *OsDof29* (**Supplementary Table S4** and **Figure S4**). Mutants of seven genes (*OsDof2*, *8*, *9*, *16*, *22*, *24*, and *26*) delayed heading by 2–5 days under both LD and SD (**Figures 4C–E**, **Table 3**, **Supplementary Table S4**, and **Figure S4**). The *Dof* family gene mutants also had effects on plant height (−14.6 to +14.1 cm under LD; −22 to +4.9 cm under SD; **Figure 4F**, **Table 3**, and **Supplementary Table S4**). Other members did not show any difference in heading date compared to the wild type (**Table 3**).

**Table 3:** Comparison of heading date and plant height traits in the CRISPR/Cas9-mediated knockout mutants of *Dof* family genes with the wild-type Zhonghua11 under long-day condition.

	PH (cm)	*P* value	HD (days)	*P* value
ZH11-WT	100.5 ± 2.1		66.9 ± 1.8	
*OsDof1*	99.0 ± 2.5	0.02	63.4 ± 1.0	4.2E−13
*OsDof2*	100.3 ± 2.6	0.43	72.0 ± 1.9	2.5E−31
*OsDof3*	98.6 ± 4.0	0.02	67.8 ± 2.4	0.07
*OsDof4*	102.4 ± 3.3	0.02	67.1 ± 2.1	0.42
*OsDof5*	107.5 ± 2.4	1.9E−09	65.9 ± 1.9	0.02
*OsDof6*	102.0 ± 4.4	0.12	66.0 ± 1.2	0.06
*OsDof7*	99.1 ± 4.7	0.15	66.9 ± 1.7	0.42
*OsDof8*	100.6 ± 2.4	0.45	68.7 ± 1.8	2.7E−06
*OsDof9*	114.6 ± 4.7	3.7E−11	69.7 ± 0.9	1.6E−05
*OsDof10*	99.2 ± 3.0	0.26	66.9 ± 0.9	0.39
*OsDof11*	113.8 ± 3.9	2.0E−14	64.5 ± 1.2	2.2E−07
*OsDof12*	100.3 ± 2.9	0.41	66.7 ± 1.4	0.29
*OsDof13*	99.6 ± 3.6	0.24	67.8 ± 1.1	0.02
*OsDof14*	105.2 ± 2.7	1.9E−06	67.0 ± 2.3	0.41
*OsDof15*	85.9 ± 4.9	7.4E−13	67.5 ± 2.1	0.06
*OsDof16*	101.3 ± 3.6	0.21	69.3 ± 1.9	1.3E−10
*OsDof17*	112.0 ± 4.5	7.4E−12	66.3 ± 1.4	0.04
*OsDof18*	105.3 ± 3.6	3.3E−05	66.4 ± 1.8	0.09
*OsDof19*	102.5 ± 3.2	0.03	67.5 ± 1.5	0.07
*OsDof20*	108.2 ± 3.5	1.0E−07	65.5 ± 2.0	0.01
*OsDof21*	110.7 ± 3.5	1.2E−06	64.2 ± 1.6	3.6E−09
*OsDof22*	102.6 ± 6.4	0.11	69.2 ± 1.7	1.1E−06
*OsDof23*	106.4 ± 3.5	3.5E−07	67.6 ± 1.6	0.02
*OsDof24*	110.7 ± 4.5	2.3E−10	68.9 ± 1.5	4.6E−09
*OsDof25*	108.7 ± 2.1	1.8E−11	67.1 ± 1.6	0.20
*OsDof26*	103.0 ± 4.3	0.02	69.5 ± 1.5	1.8E−10
*OsDof27*	109.0 ± 4.7	1.9E−07	67.6 ± 1.8	0.03
*OsDof28*	99.9 ± 4.9	0.33	67.7 ± 1.9	0.02
*OsDof29*	100.1 ± 4.3	0.56	64.4 ± 1.6	1.4E−06
*OsDof30*	102.7 ± 3.4	0.02	67.1 ± 1.8	0.26

All data are presented as the mean ± standard deviation. P values were calculated using Student's t test (n = 20 plants).

PH, plant height; HD, heading date.

### Nucleotide Diversity of *Dof* Genes

We analyzed the nucleotide diversity of *Dof* family genes in 446 wild rice and 1,612 cultivars. There were 2,822 SNPs across the entire sequence (including 2 kb of promoter and the gene body region) of the *Dof* family genes among 2,058 accessions (**Supplementary Table S5**). The pairwise nucleotide diversity ranged from 7 to 59 SNPs per kilobase for *Dof* genes (**Supplementary Table S5**). The average nucleotide diversity (*π* = 2.4 × 10^−3^) of *Dof* family genes in both *O. sativa* (*π* = 1.9 × 10^−3^) and wild rice (*π* = 2.9 × 10^−3^) was equivalent to that of the full *O. sativa* genome (*π* = 2.4 × 10^−3^). The ratio of nucleotide diversity in cultivars (*π*_c_) to nucleotide diversity in wild rice (*π*_w_) for *OsDof2*, *4*, *10*, *16*, *18*, and *24* was less than 0.5 (**Supplementary Table S5**), indicating that these genes underwent selection during domestication and genetic improvement. In particular, *OsDof24* had a very low ratio of *π*_c_/*π*_w_ of 0.17, a 5.8-fold reduction in genetic variation in *O. sativa* (**Supplementary Table S5**). Among the genes that have encountered selection, *OsDof2*, *16*, and *24* regulated heading date. Tajima's *D* of two *Dof* genes (*OsDof7* and *21*) was negative in wild rice and reached a significant level (*P* < 0.05) (**Supplementary Table S5**), suggesting that *OsDof7* and *21* have been subjected to purifying selection in wild rice. Moreover, Fu and Li's *D* value for *OsDof5*, *8*, *12*, *14*, *17*, *18*, *22*, and *29* in cultivars and *OsDof3*, *5*, and *7* in wild rice deviated significantly from neutrality (*P* < 0.05) (**Supplementary Table S5**). These results suggested that these genes have been subjected to natural selection in wild rice and artificial selection in cultivar rice.

## Discussion

### Minor Effects of *Dof* Genes on Heading Date Has Contributed to the Missing Heritability in Rice

According to the “omnigenic” model, a complex quantitative trait is controlled by a few core genes (major genes) and a large number of peripheral genes (minor genes), and the sum of minor effects across peripheral genes can far exceed the genetic contribution of core genes (Boyle et al., 2017). This statement is supported by the so-called missing heritability (Manolio et al., 2009). Much missing heritability is due to peripheral genes whose effects are too small to reach the level of genome-wide significance (Yang et al., 2015). Heading date shows high heritability in linkage mapping populations and germplasm collections. Moreover, these core heading date genes explain only a fraction of the total heritability in both GWAS and biparental mapping populations (Xue et al., 2008; Huang et al., 2010; Yan et al., 2011; Huang et al., 2012b; Yan et al., 2013; Han et al., 2016; Yano et al., 2016). There must be many genes with minor effects spread widely across the genome. It is often difficult to identify unknown genes with minor effects due to weak association signals in GWAS at the SNP level and stringent thresholds in linkage analysis. Like our *Dof* family genes, whose effects are too small to be significantly associated with heading date in GWAS (**Supplementary Figure S2**).

Heading date shows high heritability (0.75), but quantitative trait loci (QTLs) revealed by GWAS only explain 40.6% of the phenotype variation (Han et al., 2016). In this study, 22 *Dof* genes were associated with heading date *via* candidate gene-based association analysis at the haplotype level (**Supplementary Figure S4**). They all together explained about 18.4% of the variation in heading date. Moreover, mutants in 11 *Dof* genes showed a 2- to 5-day difference in heading date compared to wild-type ZH11. It is very likely that there is gene redundancy among *Dof* family genes, which results in false negatives. In addition, it is possible that some *Dof* genes in ZH11 (the transformation background genotype) are weak-functional or non-functional because ZH11 is not strongly sensitive to photoperiod (Xue et al., 2008; Yan et al., 2013; Zhang et al., 2015a). Therefore, it is understandable that some knockout mutants show no phenotype change or minor effect. But at least 11 *Dof* genes probably regulate heading date, none of which has been previously mapped, with the exception of *OsDof2*. These *Dof* genes with minor effects adequately explain part of the missing heritability. In fact, there are CCT family genes and HAP family genes in rice, and some of them also control heading date, but they have not been mapped by QTL analysis (Zhang et al., 2015b; Li et al., 2016). These minor genes together contribute to the missing heritability of heading date.

### *Dof* Family Genes Have Diverse Responses to Long-Day and Short-Day Conditions

In this study, we detected many *Dof* genes related to heading date using haplotype-based association analysis under LD (Wuhan) and SD (Hainan). Of them, 16 genes (*OsDof3*, *6*, *7*, *8*, *10*, *11*, *12*, *13*, *16*, *20*, *21*, *22*, *23*, *25*, *29*, and *30*) in the full population, four genes (*OsDof6*, *11*, *16*, and *20*) in *indica*, and six genes (*OsDof7*, *10*, *11*, *20*, *23*, and *25*) in *japonica* were commonly detected under both LD and SD (**Table 2**), which indicated that these *Dof* genes simultaneously contributed to the heritability of heading date under both conditions. One gene (*OsDof1*) in the full population, six genes (*OsDof1*, *3*, *7*, *8*, *13*, and *21*) in *indica*, and one gene (*OsDof12*) in *japonica* were detected under LD (**Table 2**), suggesting that these genes primarily function under LD. These results were supported by a previous report on *OsDof12* in which overexpressed *OsDof12* promoted rice flowering under LD, whereas overexpression of *OsDof12* had no effect under SD (Li et al., 2009). In addition, five genes (*OsDof2*, *14*, *19*, *26*, and *27*) in the full population, three genes (*OsDof2*, *10*, and *14*) in *indica*, and eight genes (*OsDof2*, *6*, *19*, *21*, *22*, *27*, *29*, and *30*) in *japonica* were detected under SD (**Table 2**), which implied that these genes primarily function under SD. Furthermore, *OsDof2* loss-of-function mutants generated by CRISPR/Cas9 showed a delay in flowering time compared with wild-type plants under both LD and SD, which was consistent with previous studies (Iwamoto et al., 2009; Iwamoto and Tagiri, 2016). However, *OsDof2* was only detected under SD in this study (**Table 2**). Taken together, *Dof* family genes have distinct responses to photoperiod.

### There Is Functional Redundancy and Differentiation Among *Dof* Family Genes

In general, functional redundancy has been reported among related genes in rice (Zhang et al., 2015b; Li et al., 2016). When knockdown or knockout mutagenesis is used for studying the function of related genes, gene redundancy can cause unclear results. In our study, there were 19 genes (**Table 2**) whose knockout mutants had heading date that were indistinguishable from wild type. Phylogenetic analysis showed that six pairs of genes, *OsDof1* and *OsDof29*, *OsDof2* and *OsDof23*, *OsDof9* and *OsDof18*, *OsDof12* and *OsDof26*, *OsDof13* and *OsDof30*, and *OsDof24* and *OsDof25*, share a very conserved gene structure outside the Dof domain (Huang et al., 2018), which is probably responsible for their similar functions. For example, knockout mutants of both *OsDof1* and *OsDof29* promoted flowering time under LD (**Table 3**). Additionally, some *Dof* genes may have functional redundancy, which causes single mutants to have wild-type phenotypes. For example, knockout mutants of both *OsDof13* and *OsDof30* did not show any change in heading date from the wild type under LD. To specify which genes are functionally redundant, double or triple mutants are needed for phenotype analysis. In addition, knockout mutants of *OsDof2*, *OsDof9*, *OsDof24*, and *OsDof26* all show delayed heading under LD, whereas mutants in their corresponding evolutionarily most closely related genes *OsDof23*, *OsDof18*, *OsDof25*, and *OsDof12* did not show any difference in heading date, suggesting a possible functional differentiation between them. It has been reported that some *Dof* genes are probably involved in stress resistance (Corrales et al., 2014). Therefore, the mutants generated in this study can also be used to evaluate stress resistance.

## Conclusion

In summary, out of 30 *Dof* genes, haplotype-based association analysis identified 22 genes associated with heading date either under long-day or short-day conditions. Favorable alleles were provided for improvement of varieties with different ecotypes. A set of knockout mutants of all 30 *Dof* genes showed that 11 and 9 *Dof* genes regulated heading date under long-day and short-day conditions, respectively. Of them, nine genes regulated heading date under both conditions. Functional redundancy might mask the phenotype of single mutants and result in missing identification of flowering *Dof* genes. The minor effects of *Dof* family genes that could not be mapped using GWAS make considerable contribution to the missing heritability of heading date in rice.

## Data Availability Statement

All datasets generated for this study are included in the article/**Supplementary Material**.

## Author Contributions

YX and YH designed the experiments. YH performed most of the experiments. YH and ZH analyzed and summed the data and drew the figures. NC, ML, and XB assisted in experiments and discussed the results. YH wrote the manuscript. YX revised the manuscript.

## Funding

This work was supported by grants from the National Key Research and Development Program of China (2016YFD0100403), the National Natural Science Foundation of China (31571751) and the Natural Science Foundation of Hubei Province, China (2015CFA006), and the Project for Applied Basis Research, Wuhan, China (2016020101010090).

## Conflict of Interest

The authors declare that the research was conducted in the absence of any commercial or financial relationships that could be construed as a potential conflict of interest.
