# Supplementary material for: Minor Effects of 11 Dof Family Genes Contribute to the Missing Heritability of Heading Date in Rice (Oryza sativa L.)
Source: Front Plant Sci. 2020 Jan 24;10:1739. doi: 10.3389/fpls.2019.01739 (PMC6993249; doi:10.3389/fpls.2019.01739)
Supplement: Supplementary file 1 [file DataSheet_1.pdf]

Table S1 CRISPR/Cas9 target sequences of the *Dof* family genes.

Gene	Target 1	Target 2
OsDof1	CGGCCTGACATGGTCCTGGA	GAGCGCCGATCATGCACTCA
OsDof2	GGACGGCTCCGACCAGCCGG	GGCTGATGTGCCAGAAGAGA
OsDof3	GCGCCGGCCCAGCTGCACAA	GCCCAGATGCTGTCCGACAC
OsDof4	GTCGTCGCCCTGCCTAAGCG	TTTACAAGGCATCAGCAGAC
OsDof5	CTTCCCGAAGAGCTTGAACC	TCGCAGACTCTGCAGGCGGC
OsDof6	GGCCCGGGAGAAGGTTGAAC	TCCTGCAACCGCCGCTGCGT
OsDof7	GATCAGCGAGTAATCGATGA	GACCCCATCCCGTGCTCCTC
OsDof8	CAGGAGTTCCAGTCCATCCC	CGCGCAGGCGCAGCAGGGCC
OsDof9	CTAGCGTTCCCGCACCACCA	TCCAGAACCCTGGGGCCTCA
OsDof10	CTCCCGACCTTCGTGTCCAC	TTCCAGCCGTAGAAGTAGTC
OsDof11	CATCATCAGTACGTGCCGTT	ATGCCCTGACGCTGTGGCTT
OsDof12	AACCTTATCAACATCGCCGG	CTTATCAGCTGAAGAGTCCT
OsDof13	ACCATCATCATCATCACGGC	CCGCTGCTGGAGGGCCGCGT
OsDof14	TCCACCTAATTGGAATAACC	ATGCACAGCACCTGCCGGTC
OsDof15	TTGTCCCTCCAAGGAGTTCT	ACGATAGGCTGCAGCACCAC
OsDof16	ACTACAGCTCCGGTGCGTCC	TTAAGCTTCAGGGCTCGTTA
OsDof17	AGGCAACCGCAGGAGCCATT	GATTTGCAGGGAGATCGCAC
OsDof18	GCAACCCCGGTGGCCCAATG	ATCATGCCGTTCCAGAACCC
OsDof19	GATCTTCCCCCCTGCCTTCC	CAGCAGCAGTCAATGATTCC
OsDof20	TCCAAGAGCGCACGCTCAGC	CCCATCCGAGGACCGTGCGG
OsDof21	CGCCGCCATCAAACCCGAAA	GGCCATCTGCGTGATGAGCC
OsDof22	CTGCTGGTGCATCTCGAACG	CGACCACCAGCAGAAGTCAT
OsDof23	TCGCCCATGTACCATCTCCA	TGAGTCATTATGTGATCGTC
OsDof24	ATTTGCTTGCCCCCGCTCCT	GTCGACCTGCGCCGTCCCAA
OsDof25	AGCCACAACCAAGAAAGCCC	AGCCCGTTCGAGTGGCCGTC
OsDof26	ACCTTGGCATCCGGCTGCAC	CAGGATGTTCAGCAGCATAG
OsDof27	ACATGCGCAGGGGTTCACCG	TCTCTTACCTGACCCCGCCT
OsDof28	TCTTCCTAGACCCTCCAAAT	CCACTCCAGTGTCTCATCGG
OsDof29	GCGTTCTCTCACCGGTTCCA	TGCAACGCGTTGGGTTGCCA
OsDof30	CTGCTGCTGGGCGGCAATCT	ACTCCCCGTTCCCAGCGCTG

Table S2 Multiple comparison of haplotype effects on heading date for associated *Dof* genes.

OsDof1	sf0137471393	sf0137471865	sf0137472496	sf0137472838	sf0137472998	sf0137473082	sf0137473101	sf0137473146	sf0137473427	sf0137473443	sf0137473485	sf0137473503	sf0137473528	sf0137473550	sf0137473637	sf0137473671	sf0137473683	sf0137474177	sf0137474233	No.*Ind*	No.*Jap*	No.*Aus*	Heading dates (d)	
																							2013WH	2013HN
Hap1	G	T	G	T	C	A	T	A	C	A	C	A	T	A	T	A	T	A	T	150	2	0	98.7±9.4 B	97.3±7.0 A
Hap2	G	T	G	T	C	A	T	A	C	A	C	A	T	G	T	A	T	A	T	86	1	13	95.9±13.5 B	94.5±8.2 AB
Hap3	A	G	G	T	G	C	T	A	T	A	C	A	G	G	C	G	T	G	C	40	0	33	114.3±11.9 A	95.7±9.4 AB
Hap4	G	G	C	A	G	C	C	T	C	A	C	G	G	G	C	A	A	G	T	2	122	0	99.4±13.9 B	94.9±11.6 AB
Hap5	G	G	C	A	G	C	C	T	C	A	T	G	G	G	C	A	A	G	T	0	13	0	113.7±11.0 A	89.9±7.1 B

OsDof2	sf0108948707	sf0108948830	sf0108948981	sf0108950087	sf0108952580	sf0108952777	sf0108952799	sf0108953081	sf0108953113	sf0108953119	sf0108953167	sf0108953205	sf0108953217	sf0108953219	sf0108953228	sf0108953251	sf0108953264	sf0108953371	No.*Ind*	No.*Jap*	No.*Aus*	Heading dates (d)
																						2013HN
Hap1	C	T	C	C	C	A	A	A	C	C	G	T	G	G	G	A	T	G	48	0	0	99.5±8.2 B
Hap2	T	T	C	C	C	A	A	A	T	C	G	T	G	G	G	A	T	G	34	0	0	92.1±8.9 BC
Hap3	T	C	G	C	C	G	G	T	C	T	T	C	A	G	G	A	C	G	14	0	42	96.1±6.1 BC
Hap4	T	T	C	C	C	A	A	A	T	C	G	T	G	G	G	A	T	G	32	3	0	95.1±6.2 BC
Hap5	T	C	C	A	G	G	G	T	C	T	T	C	A	G	G	A	C	G	87	83	0	95.5±9.6 BC
Hap6	T	C	C	A	G	G	G	T	C	T	T	C	A	G	G	A	C	G	0	11	0	84.5±4.7 D
Hap7	T	C	C	A	G	G	G	T	C	T	T	C	G	G	A	G	T	A	0	11	0	90.6±7.8 CD
Hap8	T	C	C	A	G	G	G	T	C	T	T	C	A	A	G	A	C	G	3	11	0	99.4±6.5 B
Hap9	T	C	C	A	G	G	G	T	C	T	T	C	A	A	G	A	C	G	0	25	0	107.3±5.1 A

OsDof6	sf0131849949	sf0131851306	sf0131851378	sf0131851393	sf0131851424	sf0131851664	sf0131851727	sf0131851928	sf0131852037	sf0131852045	sf0131852059	sf0131852137	sf0131852153	sf0131852180	sf0131852194	sf0131852234	sf0131852341	sf0131852546	No.*Ind*	No.*Jap*	*No.Aus*	Heading dates (d)	
																						2013WH	2013HN
Hap1	G	A	A	C	G	A	C	A	G	C	C	C	T	C	A	A	A	C	40	0	0	116.5 ±18.8 A	93.6 ±10.5 BCD
Hap2	G	T	A	C	A	G	C	A	G	C	C	C	T	C	A	A	A	C	58	12	0	99.2 ±9.9 B	99.3 ±9.4 AB
Hap3	G	T	A	C	A	G	C	A	G	C	C	C	T	C	A	A	A	T	110	1	0	98.3 ±9.9 B	98.8 ±7.3 ABC
Hap4	G	T	A	T	G	G	T	A	G	T	C	C	T	A	A	A	A	C	28	0	0	86.3 ±10.9 C	92.6 ±6.8 BCD
Hap5	T	T	G	C	G	A	C	G	T	C	T	C	C	C	G	G	G	C	43	11	0	103.8 ±12.5 B	94.9 ±6.1 BCD
Hap6	T	T	G	C	G	A	C	G	T	C	T	C	C	C	G	G	G	C	0	23	0	99.5 ±3.3 B	105.1 ±4.5 A
Hap7	T	T	G	C	G	A	C	G	T	C	T	T	C	C	G	G	G	C	0	11	0	117.3 ±8.4 A	97.5 ± 13.2 BC
Hap8	T	T	G	C	G	A	C	G	T	C	T	C	C	C	G	G	G	C	5	95	0	93.4 ±17.1 BC	87.9 ±7.9 D
Hap9	G	A	A	C	G	A	C	A	G	C	C	C	T	C	A	A	A	C	0	0	10	98.7 ±3.8 B	94.6 ±2.9 BCD
Hap10	G	A	A	C	G	A	C	A	G	C	C	C	T	C	A	A	A	C	0	0	10	98.3 ±4.1 B	89.3 ±5.1 D
Hap11	G	T	A	C	G	A	C	A	G	C	C	C	T	C	A	A	A	C	0	0	18	98.7 ±3.6 B	91.1 ±6.7 CD

OsDof7	sf0229232715	sf0229232760	sf0229233221	sf0229234278	sf0229234430	sf0229234475	sf0229234571	sf0229234971	sf0229235425	sf0229236127	sf0229236178	sf0229236416	sf0229236796	sf0229237308	sf0229237404	sf0229237514	sf0229237552	sf0229237606	No.*Ind*	No.*Jap*	*No.Aus*	Heading dates (d)	
																						2013WH	2013HN
Hap1	C	T	G	G	G	A	T	T	A	G	C	C	C	T	A	A	C	T	108	0	0	93.8 ±16.0 CD	93.7 ±8.2 B
Hap2	C	T	G	G	G	A	T	T	A	A	C	C	C	T	A	A	C	T	38	0	0	106.8 ±13.5 B	99.1 ±6.4 AB
Hap3	C	T	G	G	G	A	T	T	A	A	C	C	C	T	A	A	C	T	38	0	0	99.6 ±15.7 BCD	99.1 ±7.8 AB
Hap4	C	T	G	A	G	A	T	T	A	G	C	C	C	T	G	A	C	T	10	0	0	103.4 ±10.8 BC	100.7 ±4.1 A
Hap5	C	T	G	G	G	A	T	G	A	G	C	C	C	T	G	A	C	T	14	0	1	99.3 ±10.9 BCD	95.8 ±5.4 AB
Hap6	C	T	G	G	A	A	C	T	G	A	C	C	C	G	G	A	C	A	35	0	0	100.2 ±8.4 BCD	99.1 ±6.6 AB
Hap7	C	T	G	G	G	A	C	T	G	A	C	C	C	G	G	A	C	A	23	0	0	125.1 ±21.0 A	93.7 ±11.5 B
Hap8	A	A	G	G	G	G	T	T	G	G	T	C	T	T	G	A	C	A	7	82	43	105.9 ±12.3 B	99.6 ±9.9 AB
Hap9	A	A	A	G	G	G	T	T	G	G	C	A	C	T	G	G	T	A	4	59	0	90.0 ±15.3 D	87.0 ±7.2 C

OsDof10	sf0208590728	sf0208590739	sf0208590790	sf0208590970	sf0208591651	sf0208591652	sf0208591670	sf0208591672	sf0208591715	sf0208591742	sf0208594440	sf0208594603	sf0208594717	sf0208594747	sf0208594809	sf0208594829	sf0208594905	sf0208595288	sf0208595692	No.*Ind*	No.*Jap*	No.*Aus*	Heading dates (d)	
																							2013WH	2013HN
Hap1	C	G	G	A	A	T	C	T	C	C	C	G	T	G	T	A	G	A	A	18	1	0	100.9±5.5 AB	104.4±4.2 A
Hap2	C	G	G	A	A	C	C	T	C	T	T	A	T	G	A	G	G	A	A	16	0	9	96.5±5.1 ABC	95.7±5.3 BC
Hap3	T	C	G	A	G	C	C	G	C	C	T	A	T	G	A	A	G	G	C	32	0	0	104.9±6.7 A	96.5±5.2 ABC
Hap4	C	G	A	T	G	C	C	T	C	C	T	G	A	T	T	A	A	A	A	26	0	0	98.8±3.0 ABC	99.5±6.4 AB
Hap5	C	G	A	T	G	C	C	T	C	C	T	G	A	T	T	A	A	A	A	18	1	0	91.6±3.7 C	95.1±7.1 BCD
Hap6	C	G	A	T	G	C	C	T	C	C	T	G	A	T	T	A	A	A	A	13	1	0	96.7±3.1 ABC	100.1±4.6 AB
Hap7	C	G	G	A	G	C	C	G	T	C	T	G	T	G	T	G	G	A	A	70	55	0	99.9±10.2 ABC	98.0±10.4 AB
Hap8	C	G	G	A	G	C	C	G	T	C	T	G	T	G	T	G	G	A	A	1	11	0	99.6±2.8 ABC	103.7±5.4 A
Hap9	C	G	G	A	G	C	C	G	T	C	T	G	T	G	T	G	A	A	A	0	39	0	93.6±6.4 BC	87.8±5.3 DE
Hap10	C	G	G	A	G	C	T	G	T	C	T	G	T	G	T	G	A	A	A	0	14	0	81.1±12.6 D	85.2±6.2 E
Hap11	C	G	G	A	G	C	C	T	C	C	T	G	T	G	A	G	G	A	A	7	0	26	99.0±3.0 ABC	89.5±5.1 CDE

OsDof11	sf0321596278	sf0321596516	sf0321596517	sf0321596627	sf0321596637	sf0321596648	sf0321596689	sf0321596759	sf0321596772	sf0321596827	sf0321597360	sf0321597368	sf0321597385	sf0321597452	sf0321597460	sf0321597483	sf0321598123	sf0321598178	No.*Ind*	No.*Jap*	No.*Aus*	Heading dates (d)	
																						2013WH	2013HN
Hap1	T	C	C	C	A	A	C	C	G	C	T	C	C	G	T	C	C	C	10	0	0	100.0±10.8 AB	98.9±7.5 A
Hap2	T	C	C	C	A	A	C	C	G	C	T	C	C	G	T	C	T	C	119	0	0	100.9±11.3 AB	99.2±7.1 AB
Hap3	A	T	T	T	G	G	C	C	A	C	C	C	G	A	C	C	C	C	67	0	0	91.5±9.1 B	94.2±8.5 ABC
Hap4	A	T	C	T	G	G	C	C	A	C	C	T	G	A	C	C	C	A	64	0	11	109.8±16.4 A	91.8±8.3 BCD
Hap5	A	T	C	C	G	G	T	T	A	A	C	C	C	G	T	C	T	C	9	123	0	102.5±13.4 AB	95.2±11.1 ABC
Hap6	A	T	C	C	G	G	T	T	A	A	C	C	C	G	T	T	T	C	0	18	0	78.2±7.1 C	84.4±6.9 D
Hap7	A	C	C	C	A	G	C	C	G	C	C	C	G	A	C	C	T	C	0	0	11	97.2±4.1 B	92.8±8.9 ABC
Hap8	A	C	C	C	G	G	T	T	A	C	C	C	C	G	C	C	C	C	0	0	20	99.2±2.8 AB	88.9±4.7 CD

OsDof13	sf0323470657	sf0323473338	sf0323474234	sf0323474416	sf0323474491	sf0323474495	sf0323474573	sf0323474585	sf0323474790	sf0323474893	sf0323474896	sf0323474958	sf0323475001	sf0323475120	sf0323475263	sf0323475393	sf0323475515	sf0323475535	sf0323475556	No.*Ind*	No.*Jap*	No.*Aus*	Heading dates (d)	
																							2013WH	2013HN
Hap1	A	C	C	T	T	G	G	G	T	C	C	C	C	C	C	G	T	G	T	2	118	0	96.6±12.2 BC	92.5±10.8 C
Hap2	A	C	C	T	T	G	G	G	T	C	T	C	C	C	C	G	T	G	T	1	16	0	106.4±10.7 A	102.8±10.6 A
Hap3	T	C	A	T	T	A	A	G	A	C	T	C	T	T	T	G	C	A	T	56	0	0	89.5±9.5 C	94.3±7.5 BC
Hap4	T	A	C	A	C	G	G	A	T	T	C	T	C	C	C	A	T	G	T	18	0	0	103.1±8.8 AB	100.1±6.4 AB
Hap5	T	A	C	A	C	G	G	A	T	T	C	T	C	C	C	A	T	G	T	111	0	0	101.9±11.1 AB	98.7±6.7 AB
Hap6	T	A	C	A	C	G	G	G	T	T	C	T	C	C	C	A	T	G	T	53	0	6	106.6±12.8 A	92.2±7.1 C
Hap7	T	C	C	A	C	G	G	G	T	T	C	T	C	C	C	A	T	G	T	16	0	0	100.7±5.2 AB	98.0±6.9 ABC
Hap8	T	C	C	A	T	G	G	G	T	C	C	C	C	C	C	G	C	G	C	0	0	32	99.3±3.7 AB	91.9±6.3 C

OsDof16	sf0334446637	sf0334446866	sf0334446951	sf0334446975	sf0334446977	sf0334447510	sf0334447535	sf0334447571	sf0334447686	sf0334447825	sf0334449076	sf0334449600	sf0334449684	No.*Ind*	No.*Jap*	No.Aus	Heading dates (d)	
																	2013WH	2013HN
Hap1	A	C	G	C	G	G	T	A	G	G	G	A	A	228	2	7	97.6±11.2 B	96.1±8.2 AB
Hap2	A	C	G	C	G	G	C	C	G	G	G	A	A	26	0	0	108.3±15.0 A	90.2±7.5 C
Hap3	A	T	G	T	T	T	C	C	A	T	C	G	C	12	102	0	103.9±14.3 AB	97.2±12.2 A
Hap4	A	T	A	T	T	T	C	C	A	T	C	G	C	0	50	0	90.3±13.0 C	88.6±7.7 C
Hap5	T	C	G	C	G	G	C	C	G	G	G	A	A	0	1	34	99.1±3.8 B	91.6±6.6 BC

OsDof21	sf0707603631	sf0707603677	sf0707603687	sf0707603750	sf0707603912	sf0707603938	sf0707604166	sf0707604212	sf0707605272	sf0707605694	sf0707605704	sf0707605741	sf0707605791	sf0707606178	sf0707606225	sf0707606315	sf0707606407	sf0707606561	sf0707606636	No.*Ind*	No.*Jap*	No.*Aus*	Heading dates (d)	
																							2013WH	2013HN
Hap1	C	T	C	C	T	A	G	C	C	G	C	C	G	A	C	C	C	A	C	12	0	0	81.5 ±9.0 BC	86.2 ±4.1 DE
Hap2	C	T	C	C	T	A	G	C	T	G	C	C	G	A	C	C	C	A	C	242	3	3	100.1 ±12.1 A	97.0 ±7.8 BC
Hap3	C	T	T	C	T	A	G	C	T	G	C	C	G	A	C	C	C	A	C	11	0	0	109.0 ±15.4 A	98.7 ±8.4 BC
Hap4	G	C	C	C	C	G	G	C	T	A	C	C	A	G	T	C	C	T	C	0	12	0	75.9 ±11.9 C	83.2 ±7.2 E
Hap5	G	C	C	C	C	G	G	C	T	G	C	C	A	G	T	C	C	T	C	7	72	18	99.3 ±14.5 A	90.5 ±9.0 CDE
Hap6	G	C	C	C	C	G	G	C	T	G	C	C	A	G	T	T	C	T	C	0	16	0	103.1 ±5.5 A	108.2 ±6.4 A
Hap7	G	C	C	C	C	G	G	C	T	G	T	C	A	G	T	C	C	T	C	0	14	0	94.0 ±13.7 AB	87.3 ±2.7 DE
Hap8	C	C	C	T	T	A	G	T	T	G	C	C	G	A	C	C	C	T	T	4	14	0	105.9 ±10.1 A	103.2 ±5.1 AB
Hap9	G	C	C	C	C	G	G	C	T	G	C	C	A	G	T	C	C	T	C	0	1	12	96.7 ±3.5 A	87.7 ±4.7 DE
Hap10	C	C	C	C	T	A	C	C	T	G	C	T	G	A	C	C	T	T	C	0	0	10	100.3 ±2.9 A	94.0 ±5.6 CD

OsDof25	sf0918232249	sf0918232616	sf0918232689	sf0918232791	sf0918232840	sf0918232846	sf0918232909	sf0918233028	sf0918233089	sf0918233133	sf0918233224	sf0918233274	sf0918233341	sf0918233349	sf0918233415	sf0918233685	sf0918234363	sf0918234383	sf0918234449	No.*Ind*	No.*Jap*	No.*Aus*	Heading dates (d)	
																							2013WH	2013HN
Hap1	C	G	T	T	C	C	G	A	C	C	T	C	A	C	A	G	C	C	A	180	0	0	100.2 ±11.5 A	97.0 ±8.2 B
Hap2	C	G	G	C	C	T	A	G	T	T	G	A	C	T	G	T	T	T	C	51	0	0	102.3 ±14.9 A	94.7 ±7.2 B
Hap3	C	C	G	C	C	C	A	G	T	T	G	A	C	T	A	T	T	T	C	0	17	0	108.1 ±5.1 A	108.9 ±7.6 A
Hap4	C	G	G	C	C	C	A	G	T	T	G	A	C	T	A	T	T	T	C	8	53	0	85.2 ±14.1 B	87.1 ±7.7 D
Hap5	C	G	G	C	C	C	A	G	T	T	G	A	C	T	A	T	T	T	C	3	41	5	105.5 ±15.9 A	88.9 ±9.4 CD
Hap6	T	G	G	C	C	C	A	G	T	T	G	A	C	T	A	T	T	T	C	0	29	0	100.3 ±5.9 A	104.7 ±6.8 A
Hap7	C	G	T	T	T	C	G	A	C	C	T	C	A	C	A	G	C	C	A	9	1	31	99.6 ±4.1 A	92.7 ±6.1 BC

OsDof29	sf0521491795	sf0521491835	sf0521491933	sf0521492190	sf0521492224	sf0521492411	sf0521492446	sf0521492587	sf0521492602	sf0521492634	sf0521492687	sf0521492697	sf0521492743	sf0521492786	sf0521492955	sf0521493102	sf0521493567	sf0521493722	sf0521493846	No.*Ind*	No.*Jap*	No.*Aus*	Heading dates (d)	
																							2013WH	2013HN
Hap1	G	T	A	A	A	A	A	C	G	G	G	A	T	A	A	G	T	C	C	189	0	14	103.4 ±12.8 A	96.2 ±8.1 B
Hap2	G	T	T	A	A	A	A	C	G	G	G	A	T	A	A	G	T	C	C	37	0	0	95.3 ±14.3 AB	97.8 ±7.2 B
Hap3	G	T	A	G	G	G	T	C	G	G	A	A	T	T	G	G	T	A	C	36	1	0	92.3 ±11.3 AB	93.2 ±8.3 BC
Hap4	A	T	A	G	G	G	T	C	G	A	G	A	C	A	G	T	G	C	T	0	39	0	102.8 ±7.8 A	105.0 ±6.8 A
Hap5	G	T	A	G	G	G	T	C	G	A	G	A	C	A	G	T	G	C	T	0	10	3	97.9 ±13.0 AB	96.5 ±11.0 B
Hap6	G	T	A	G	G	G	T	T	G	A	G	A	C	A	G	T	G	C	T	2	67	0	100.3 ±15.2 AB	89.0 ±8.3 C
Hap7	G	C	A	G	G	G	T	C	T	A	G	T	C	A	G	T	G	C	T	0	13	0	87.6 ±13.2 B	86.4 ±7.6 C

OsDof30	sf1224691483	sf1224691514	sf1224691574	sf1224691625	sf1224692233	sf1224692375	sf1224692386	sf1224692494	sf1224692513	sf1224692580	sf1224692622	sf1224692675	sf1224692723	sf1224692835	sf1224692917	sf1224692954	sf1224692977	sf1224693038	No.*Ind*	No.*Jap*	No.*Aus*	Heading dates (d)	
																						2013WH	2013HN
Hap1	G	G	C	T	A	A	C	A	A	G	G	T	C	G	C	G	A	C	11	0	44	92.5 ±4.1 BC	90.5 ±6.1 AB
Hap2	G	G	T	C	G	G	C	G	G	G	G	T	T	G	C	G	G	T	267	33	0	100.9 ±12.3 AB	97.5 ±9.2 A
Hap3	G	A	C	C	G	G	A	G	A	A	A	C	C	C	T	A	G	C	0	78	0	97.0 ±17.0 AB	87.9 ±7.5 BC
Hap4	A	A	C	C	G	G	C	G	A	A	A	C	C	C	T	A	G	C	0	11	0	76.9 ±7.8 C	82.0 ±4.3 C
Hap5	G	A	C	C	G	G	C	G	A	A	A	C	C	C	T	A	G	C	2	28	0	110.1 ±8.6 A	97.1 ±10.5 A

Table S3 The rates of homozygous mutation in T_0 transgenic plants.

Genes	Host cultivar	No. of transgenic events	No. of events with homozygous mutations	Homozygous mutation rate (%)
OsDof1	Zhonghua 11	73	7	9.6
OsDof2	Zhonghua 11	22	4	18.2
OsDof3	Zhonghua 11	70	8	11.4
OsDof4	Zhonghua 11	48	7	14.6
OsDof5	Zhonghua 11	49	6	12.2
OsDof6	Zhonghua 11	72	6	8.3
OsDof7	Zhonghua 11	36	4	11.1
OsDof8	Zhonghua 11	50	7	14.0
OsDof9	Zhonghua 11	36	4	11.1
OsDof10	Zhonghua 11	70	4	5.7
OsDof11	Zhonghua 11	70	11	15.7
OsDof12	Zhonghua 11	57	15	26.3
OsDof13	Zhonghua 11	39	7	17.9
OsDof14	Zhonghua 11	33	5	15.2
OsDof15	Zhonghua 11	13	3	23.1
OsDof16	Zhonghua 11	22	4	18.2
OsDof17	Zhonghua 11	26	4	15.4
OsDof18	Zhonghua 11	13	3	23.1
OsDof19	Zhonghua 11	39	6	15.4
OsDof20	Zhonghua 11	69	11	15.9
OsDof21	Zhonghua 11	30	4	13.3
OsDof22	Zhonghua 11	50	8	16.0
OsDof23	Zhonghua 11	29	4	13.8
OsDof24	Zhonghua 11	27	5	18.5
OsDof25	Zhonghua 11	26	6	23.1
OsDof26	Zhonghua 11	23	5	21.7
OsDof27	Zhonghua 11	60	8	13.3
OsDof28	Zhonghua 11	60	9	15.0
OsDof29	Zhonghua 11	21	5	23.8
OsDof30	Zhonghua 11	32	3	9.4

Table S4 Comparison of heading date and plant height traits in the CRISPR-Cas9-mediated knockout mutants of *Dof* family genes with the wild-type Zhonghua11 under short-day condition.

	PH (cm)	*P* value	HD (d)	*P* value
ZH11-WT	74.5±4.2		55.3±1.2	
OsDof1	74.2±4.7	0.41	52.9±2.8	3.9E-07
OsDof2	74.7±5.7	0.42	59.5±2.6	1.3E-09
OsDof3	76.0±4.1	0.09	55.8±1.1	0.32
OsDof4	75.8±4.9	0.14	56.1±1.8	0.08
OsDof5	79.2±4.4	5.2E-06	55.6±2.5	0.26
OsDof6	75.1±5.0	0.26	55.9±1.4	0.21
OsDof7	71.5±4.0	1.3E-03	55.5±2.1	0.38
OsDof8	72.7±4.2	0.07	58.3±1.5	4.3E-11
OsDof9	79.4±5.0	4.3E-04	59.5±2.3	1.8E-11
OsDof10	73.5±5.2	0.29	55.0±1.2	0.32
OsDof11	67.2±5.1	1.4E-11	56.0±1.7	0.11
OsDof12	71.0±4.2	1.4E-03	54.9±0.9	0.1
OsDof13	52.5±4.2	4.8E-33	56.2±2.0	0.04
OsDof14	77.2±4.3	2.7E-03	56.1±1.6	0.06
OsDof15	67.5±3.0	3.5E-09	55.6±1.8	0.47
OsDof16	72.7±4.1	0.02	57.6±2.3	6.7E-05
OsDof17	74.9±4.0	0.31	56.2±1.3	0.16
OsDof18	70.6±3.5	5.0E-05	55.7±1.5	0.42
OsDof19	73.1±4.4	0.06	55.6±1.4	0.39
OsDof20	72.3±4.5	9.9E-03	55.4±1.0	0.36
OsDof21	68.3±4.7	1.8E-09	51.7±2.3	4.0E-12
OsDof22	62.6±4.0	1.0E-20	60.1±2.6	2.2E-11
OsDof23	74.1±3.2	0.37	55.0±1.3	0.37
OsDof24	75.3±3.0	0.25	57.1±1.4	3.6E-07
OsDof25	72.8±4.6	0.05	56.3±1.3	0.12
OsDof26	73.5±4.1	0.15	59.9±2.0	1.3E-13
OsDof27	74.9±4.8	0.34	56.2±1.2	0.11
OsDof28	73.5±3.7	0.11	55.8±1.0	0.23
OsDof29	66.5±4.0	6.5E-14	56.3±2.1	0.06
OsDof30	73.0±4.3	0.06	55.8±1.0	0.21

PH, plant height; HD, heading date; All data are presented as the mean ± standard deviation. *P* values were calculated using Student's *t*-test (n = 20 plants).

Table S5 The nucleotide diversity of *Dof* family genes in cultivars and wild rice.

Genes	SNPs	π (10^{-3})		π_c/π_w	Tajima's *D*		Fu and Li's *D*	
		C	W		C	W	C	W
OsDof1	27	0.48	0.80	0.61	-0.63	-0.90	-2.33	-0.58
OsDof2	148	1.61	3.91	0.41	0.23	-0.94	-1.23	-0.78
OsDof3	79	1.40	2.28	0.61	0.31	-1.42	-0.66	1.64*
OsDof4	193	2.04	4.67	0.44	1.30	-1.02	0.14	0.16
OsDof5	31	0.41	0.75	0.55	-1.12	-1.58	-5.09**	1.60*
OsDof6	51	1.59	1.75	0.91	1.90	-1.66	0.26	0.42
OsDof7	85	1.00	1.16	0.86	-0.61	-1.77*	-1.76	1.70*
OsDof8	66	1.55	2.38	0.65	-0.66	-0.94	-5.01*	1.42
OsDof9	60	1.15	1.51	0.76	1.00	-1.08	-1.67	1.48
OsDof10	242	3.48	8.59	0.40	-1.65	-1.38	-2.10	-0.38
OsDof11	70	2.45	2.87	0.85	0.17	-1.05	-1.67	0.77
OsDof12	80	0.82	1.31	0.63	-1.46	-1.69	-3.34*	1.39
OsDof13	202	2.23	4.38	0.51	-1.26	-1.38	0.32	0.56
OsDof14	40	0.79	1.55	0.51	-1.07	-1.09	1.55*	-0.63
OsDof15	74	1.67	2.74	0.61	-0.68	-1.48	-2.09	0.05
OsDof16	43	0.72	1.67	0.43	-0.99	-0.83	0.71	0.84
OsDof17	101	2.18	2.77	0.79	-0.05	-1.63	-2.95*	0.73
OsDof18	59	0.46	1.78	0.26	-1.63	-1.40	-2.58*	1.21
OsDof19	69	1.27	1.70	0.75	-1.83	-1.43	-0.37	0.70
OsDof20	168	4.47	5.19	0.86	-0.53	-1.30	0.32	0.46
OsDof21	125	3.37	2.76	1.22	-0.40	-1.89*	0.23	0.61
OsDof22	60	1.60	2.33	0.69	-0.20	-1.19	-2.40*	-0.47
OsDof23	86	1.19	1.46	0.82	-0.05	-1.56	0.12	0.43
OsDof24	86	0.70	4.04	0.17	-0.89	-0.40	-2.17	0.70
OsDof25	128	4.05	5.80	0.70	-0.12	-1.20	-1.04	-1.02
OsDof26	100	2.31	3.37	0.69	1.68	0.07	-0.19	-0.18
OsDof27	91	2.55	3.27	0.78	-0.59	-1.68	0.41	0.16
OsDof28	89	2.39	3.56	0.67	-1.04	-1.50	-0.17	0.00
OsDof29	94	4.32	4.08	1.06	0.34	-1.41	-3.71**	-0.77
OsDof30	75	1.87	2.74	0.68	0.16	-1.27	-0.49	-0.15

SNPs were extracted from the genomic region included 2-kb of promoter and the gene body region. Nucleotide diversity (π), Tajima's *D* and Fu and Li's *D* statistic were calculated both in cultivars and wild rice (*O. rufipogon*). π_c and π_w indicate π in cultivars and π in wild rice respectively. *$P < 0.05$, **$P < 0.02$.

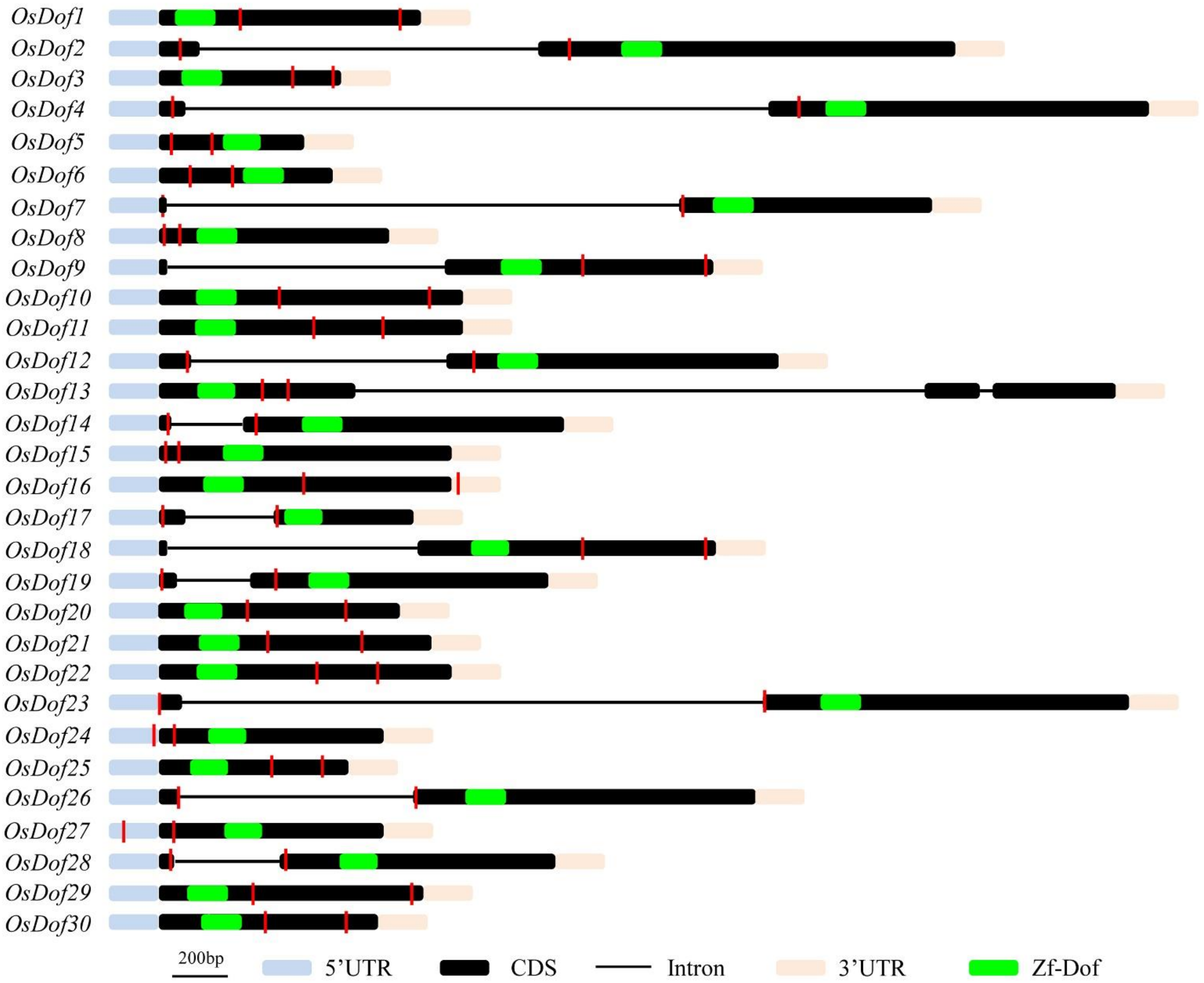

Figure S1 Structures of *Dof* family genes in rice.

The red line indicated the CRISPR/Cas9 target sites used in CRISPR/Cas9.

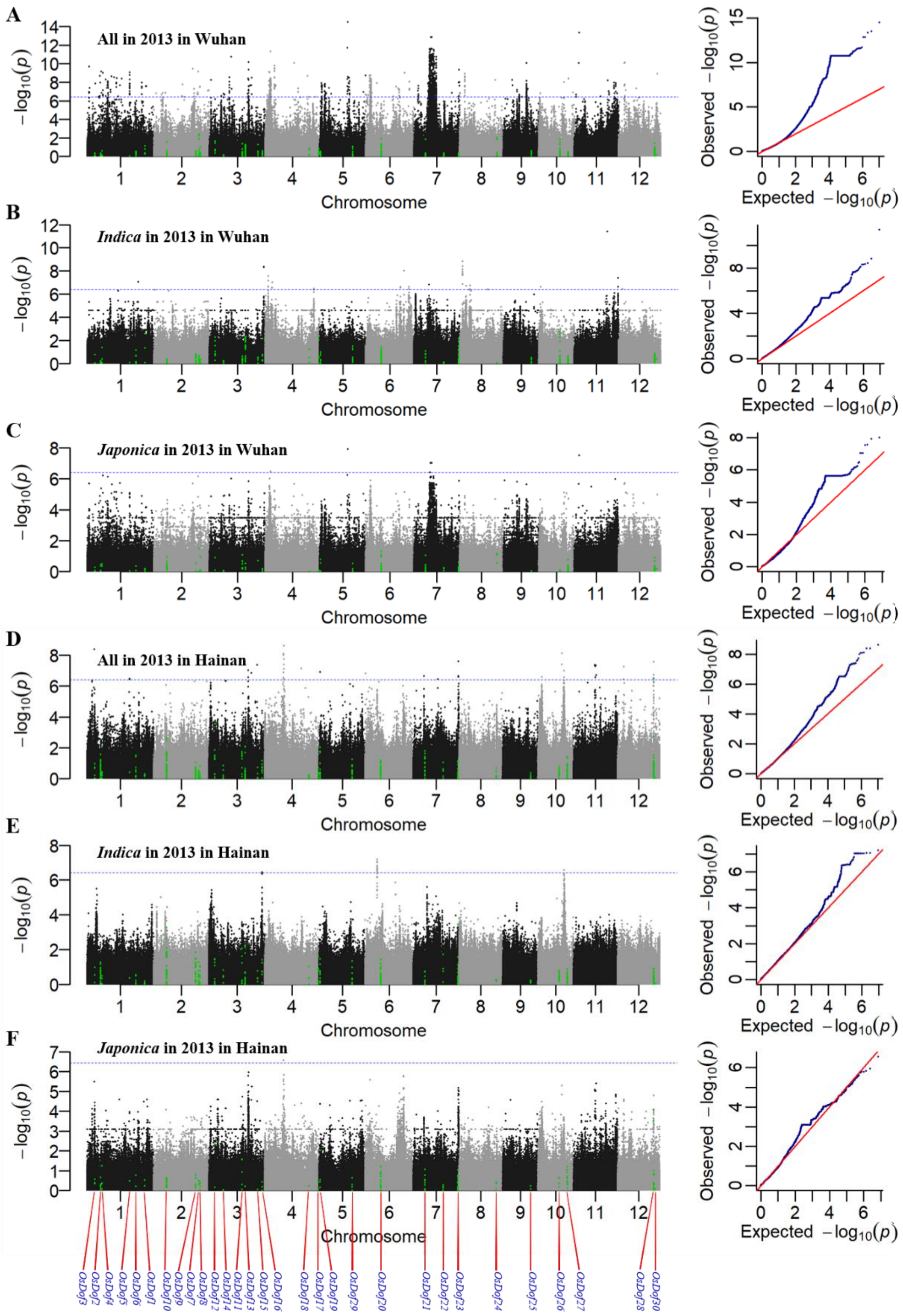

Figure S2 Genome-wide *P*-values and quantile-quantile plots from linear mixed model (LMM) for heading date in all accessions, *indica* and *japonica* in 2013 Wuhan and 2013 Hainan.

The horizontal dashed line indicates the genome-wide significance threshold ($P = 3.8E-07$). Green dots represent the *P*-values of SNPs of 30 *Dof* family genes.

CRISPR-*OsDof1*:

```
-576        GCCCGGTCCGGCCTGA----------------------------------------------------TCATG
-546        GCCCGGTCCGGCCTGACATGGTCC-T----------------------------GTGCATGATCGGCGCTCCTGATCATG
+1 T, -1 A  GCCCGGTCCGGCCTGACATGGTCCTTGGAAGGCATG---CCTCCATG-GTGCATGATCGGCGCTCCTGATCATG
WT          GCCCGGTCCGGCCTGACATGGTCC-TGGAAGGCATG---CCTCCATGAGTGCATGATCGGCGCTCCTGATCATG
                    CGGCCTGACATGGTCC-TGGA                TGAGTGCATGATCGGCGCTC
```

CRISPR-*OsDof2*:

```
-1 G, +1 A  CGGCGGCGGACGGCTCCGACCA-C-CGGAGGCG----------GTGACGGCTGATGTGCCAGAAGAAGAAGG
+1 T, +1 C  CGGCGGCGGACGGCTCCGACCAGCTCGGAGGCG----------GTGACGGCTGATGTGCCAGAAGCAGAAGG
+1 T, +1 A  CGGCGGCGGACGGCTCCGACCAGCTCGGAGGCG----------GTGACGGCTGATGTGCCAGAAGAAGAAGG
+1 A, -6    CGGCGGCGGACGGCTCCGACCAGCACGGAGGCG----------GTGACGGCTGATGTGC-------AGAAGG
WT          CGGCGGCGGACGGCTCCGACCAGC-CGGAGGCG----------GTGACGGCTGATGTGCCAGAAG-AGAAGG
                   GGACGGCTCCGACCAGC-CGG                    GGCTGATGTGCCAGAAG-AGA
```

CRISPR-*OsDof3*:

```
-213        TGG----------------------------------------------------------------------GGT
+1 T, -116  TGGCGGCCGT----GCTCGCGCCGGCCCAGCTGCATC---------------- GGACAGCATCTGGGCCATGGGGT
WT          TGGCGGCCGT----GCTCGCGCCGGCCCAGCTGCA-CAACG---CGGTGTCGGACAGCATCTGGGCCATGGGGT
                              GCGCCGGCCCAGCTGCA-CAA          GTGTCGGACAGCATCTGGGC
```

CRISPR-*OsDof4*:

```
-2371       GCCGCTATGGCCGCG---------------------------------------------------------- CGGAT
-2366       GCCGCTATGGCCGCGCT---------------------------------------------------- GACCGGAT
-2367       GCCGCTATGGCCGCGC----------------------------------------------------- GACCGGAT
WT          GCCGCTATGGCCGCGCTTAGGCAGGGCGACGACCCCGCC--------ATGATTTACAAGGCATCAGCAGACCGGAT
                         CGCTTAGGCAGGGCGACGAC                   TTTACAAGGCATCAGCAGAC
```

CRISPR-*OsDof5*:

```
-151, -9    CCGGGT-------------------------------------------GGCGGGCGGTGGCGGTGGC----------GG
WT          CCGGGTTCAAGCTCTTCGGGAAGGT---CGTCGCAGACTCTGCAGGCGGCGGGCGGTGGCGGTGGCGGAGGAGGGGG
               GGTTCAAGCTCTTCGGGAAG         TCGCAGACTCTGCAGGCGGC
```

CRISPR-*OsDof6*:

```
-150        TCGTCGTGGCCCGGGAG--------------------------------------------- CGGCGGTTGCAGGACTCGG
-2T         TCGTCGTGGCCCGGGAGAAGG---GAACCGGAG----------GCAGCCGACG-CAGCGGCGGTTGCAGGACTCGG
+1 G, +1 T  TCGTCGTGGCCCGGGAGAAGGTTGGAACCGGAG----------GCAGCCGACGTCAGCGGCGGTTGCAGGACTCGG
WT          TCGTCGTGGCCCGGGAGAAGGTT-GAACCGGAG---------- GCAGCCGACG-CAGCGGCGGTTGCAGGACTCGG
                   GGCCCGGGAGAAGGTT-GAAC                          ACG-CAGCGGCGGTTGCAGGA
```

CRISPR-*OsDof7*:

```
-1952       ATTAAGATCAGCGAGTAATCGA--------------------------- GAGCACGGGATGGGGTCGTC
-1948, -1 G ATTAAGATCAGCGAGTAATC------------------------- CCCGAG-AGCACGGGATGGGGTCGTC
WT          ATTAAGATCAGCGAGTAATCGATGATGGCAGG--------TCCCCGAGGAGCACGGGATGGGGTCGTC
                 GATCAGCGAGTAATCGATGA                   GAGGAGCACGGGATGGGGTC
```

CRISPR-*OsDof8*:

```
-111        CTC------------------------------------------------------------------------- GGG
-1 T, +1 T  CTCATGCAGGAGTTCCAGTCCA--CCCGG------GCGCGCGCAGGCGCAGCAGGTGCCCGGCGTCGCGGTGCGGCGGG
+1 T, +1 A  CTCATGCAGGAGTTCCAGTCCATTCCCGG------GCGCGCGCAGGCGCAGCAGGAGCCCGGCGTCGCGGTGCGGCGGG
WT          CTCATGCAGGAGTTCCAGTCCAT-CCCGG------GCGCGCGCAGGCGCAGCAGG-GCCCGGCGTCGCGGTGCGGCGGG
                  CAGGAGTTCCAGTCCAT-CCC           CGCGCAGGCGCAGCAGG-GCC
```

CRISPR-*OsDof9*:

```
-523        CTCAACCTAGCGTTCCCGCACC-------------------------------GGCCCCAGGGTTCTGGAATAGCA
WT          CTCAACCTAGCGTTCCCGCACCACCACGGCC----------- TGGCCATGAGGCCCCAGGGTTCTGGAATAGCA
                  CTAGCGTTCCCGCACCACCA                     TGAGGCCCCAGGGTTCTGGA
```

CRISPR-*OsDof10*:

```
-526        CGTGCTCCCGACCTTCGTGTC---------------------------------- TACTTCTACGGCTGGAACAGCG
WT          CGTGCTCCCGACCTTCGTGTCCACCGGGT----------------AGGCCGGACTACTTCTACGGCTGGAACAGCG
                CTCCCGACCTTCGTGTCCAC                          GACTACTTCTACGGCTGGAA
```

CRISPR-*OsDof11*:

```
-146, +1 G   GGGCATCATCAGTA--------------------------AGCAGCA---CCCAAGTCCACAGCGTCAGGGCATGCTG
+1 G         GGGCATCATCAGTACGTGCCGTTCGG------ CTGCAGCAGCA------CCCAAGGCCACAGCGTCAGGGCATGCTG
WT           GGGCATCATCAGTACGTGCCGTTCGG------ CTGCAGCAGCA------CCCAA-GCCACAGCGTCAGGGCATGCTG
                CATCATCAGTACGTGCCGTT                                   AA-GCCACAGCGTCAGGGCAT
```

CRISPR-*OsDof12*:

```
-1065        CGCCTGCGCCGCCG-------------------------------------------------ACTCTTCAGCTGATAAGAAC
-17, -1 A    CGCCTGCG------------------AAGGTTGGTGT----------------ACACCGAGG--CTCTTCAGCTGATAAGAAC
+1 T, +1 T   CGCCTGCGCCGCCGTGCGATGTTGATAAGGTTGGTGT--------------- ACACCGAGGTACTCTTCAGCTGATAAGAAC
+1 A, +1 G   CGCCTGCGCCGCCGAGCGATGTTGATAAGGTTGGTGT--------------- ACACCGAGGGACTCTTCAGCTGATAAGAAC
WT           CGCCTGCGCCGCCG-GCGATGTTGATAAGGTTGGTGT--------------- ACACCGAGG-ACTCTTCAGCTGATAAGAAC
                         CCG-GCGATGTTGATAAGGTT                             AGG-ACTCTTCAGCTGATAAG
```

CRISPR-*OsDof13*:

```
+1 T         TCATCACCATCATCATCATCACGGCGG--------------- CGGCGCCGCTGCTGGAGGGCCGTCGTCG
WT           TCATCACCATCATCATCATCACGGCGG--------------- CGGCGCCGCTGCTGGAGGGCCG-CGTCG
                  ACCATCATCATCATCACGGC                        CCGCTGCTGGAGGGCCG-CGT
```

CRISPR-*OsDof14*:

```
-321         AGATCCACCTAATTGGAAT-----------------------------------------GTCGG
-1 A, +1 T   AGATCCACCTAATTGGAAT--ACCAGGTA---------- GGCGATGCACAGCACCTGCCGTGTCGG
+1 C, +1 T   AGATCCACCTAATTGGAATACACCAGGTA----------GGCGATGCACAGCACCTGCCGTGTCGG
WT           AGATCCACCTAATTGGAATA-ACCAGGTA---------- GGCGATGCACAGCACCTGCCG-GTCGG
                TCCACCTAATTGGAATA-ACC                   ATGCACAGCACCTGCCG-GTC
```

CRISPR-*OsDof15*:

```
+1 A, -9     TCCAAGAAACTCCTTGGAGGGACAACCATG-------TCCCTGCCGGTG----------CCTATCGTGTC
-4, -6       TCCAAG-AATTG----GAGGGACAACCATG-------TCCCTGCCGGTG-------CAGCCTATCGTGTC
+1 T         TCCAAG-AACTCCTTGGAGGGACAACCATG-------TCCCTGCCGGTGTGTGCTGCAGCCTATCGTGTC
WT           TCCAAG-AACTCCTTGGAGGGACAACCATG-------TCCCTGCCGGTG-GTGCTGCAGCCTATCGTGTC
                 AG-AACTCCTTGGAGGGACAA                    GTG-GTGCTGCAGCCTATCGT
```

CRISPR-*OsDof16*:

```
-1 T, +1 T   CGATCACTACAGCTCCGGTGCG-CCAGGC----------CCCTAATTCGAGCCCTGAAGCTTAATTT
-1 T, +1 A   CGATCACTACAGCTCCGGTGCG-CCAGGC----------CCCTAAACGAGCCCTGAAGCTTAATTT
WT           CGATCACTACAGCTCCGGTGCGTCCAGGC----------CCCTAA-CGAGCCCTGAAGCTTAATTT
                  ACTACAGCTCCGGTGCGTCC                    TAA-CGAGCCCTGAAGCTTAA
```

CRISPR-*OsDof17*:

```
+1 T, +1 A   ACCAAATTGGCTCCTGCGGTTGCCTCCT--------TTTGATTTGCAGGGAGATCGACACGGGG---------CGCAGGGTCTC
+1 G, +1 A   ACCAAATGGGCTCCTGCGGTTGCCTCCT--------TTTGATTTGCAGGGAGATCGACACGGGG---------CGCAGGGTCTC
+1 T, -218   ACCAAATTGGCTCCTGCGGTTGCCTCCT--------TTTGATTTGCAGGG--------------------------------- TCTC
WT           ACCAAAT-GGCTCCTGCGGTTGCCTCCT--------TTTGATTTGCAGGGAGATCG-CACGGGG---------CGCAGGGTCTC
                 AAT-GGCTCCTGCGGTTGCCT               GATTTGCAGGGAGATCG-CAC
```

CRISPR-*OsDof18*:

```
-516         CGGGCTG------------------------------------------------------ TTCTGGAACGGCATGATCGG
-447         CGGGCTGCAGGCG-------TGTGCAACCCCGGTGGCCCA-----------------------TTCTGGAACGGCATGATCGG
WT           CGGGCTGCAGGCG-------TGTGCAACCCCGGTGGCCCAATGGGG---------- CGGGGTTCTGGAACGGCATGATCGG
                                    GCAACCCCGGTGGCCCAATG                  GGGTTCTGGAACGGCATGAT
```

CRISPR-*OsDof19*:

```
-2, +1 T     ATGATCTTCCCCCCTGC--TCCTGGATT----------TCTGCAGCAGCAGTCAATGATTTCCGGGC
-1 T         ATGATCTTCCCCCCTGCC-TCCTGGATT----------TCTGCAGCAGCAGTCAATGA-TTCCGGGC
WT           ATGATCTTCCCCCCTGCCTTCCTGGATT----------TCTGCAGCAGCAGTCAATGA-TTCCGGGC
               GATCTTCCCCCCTGCCTTCC                    CAGCAGCAGTCAATGA-TTCC
```

CRISPR-*OsDof20*:

```
-422         GC------------------------------------------------------------------------------ CGA
-357         GCGACTCCAAGAGCGCACGCTC----------------- ACGGTCCTCGGATGGGCCGGCAGCGGCTGCCGCCGCCGCCGCCGCCGA
+1 T         GCGACTCCAAGAGCGCACGCTCTAGCCGG--- CGCCGCACGGTCCTCGGATGGGCCGGCAGCGGCTGCCGCCGCCGCCGCCGCCGA
WT           GCGACTCCAAGAGCGCACGCTC-AGCCGG--- CGCCGCACGGTCCTCGGATGGGCCGGCAGCGGCTGCCGCCGCCGCCGCCGCCGA
                  TCCAAGAGCGCACGCTC-AGC            CCGCACGGTCCTCGGATGGG
```

CRISPR-*OsDof21*:

```
+1 A, -17    TCCCTTTACGGGTTTGATGGCGGCGGCGG-----------------TCCGCC----------------GGCCTCGG
+1 A, +1 T   TCCCTTTACGGGTTTGATGGCGGCGGCGG-----------------TCCGCCGGGCTTCATCACGCAGATGGCCTCGG
WT           TCCCTTT-CGGGTTTGATGGCGGCGGCGG-----------------TCCGCCGGGC-TCATCACGCAGATGGCCTCGG
                 TTT-CGGGTTTGATGGCGGCG                              GGC-TCATCACGCAGATGGCC
```

CRISPR-*OsDof22*:

```
-233         GCCGCG-------------------------------------------------------GGC
+1 G, +1 T   GCCGCGTGTCGAGATGCACCAGCAGCATC------------CCGGCGACCACCAGCAGAAGTTCATCGGC
+1 T, +1 T   GCCGCGTTTCGAGATGCACCAGCAGCATC------------CCGGCGACCACCAGCAGAAGTTCATCGGC
WT           GCCGCGT-TCGAGATGCACCAGCAGCATC------------CCGGCGACCACCAGCAGAAGT-CATCGGC
                 CGT-TCGAGATGCACCAGCAG                    CGACCACCAGCAGAAGT-CAT
```

CRISPR-*OsDof23*:

```
-2, -3       TCTCTCGCCCATGTACCA---TCCATGG---------------TTACTGAGTCATTATGT---C-GTCAGGA
+1 T, +1 A   TCTCTCGCCCATGTACCATCTTCCATGG---------------TTACTGAGTCATTATGTGATCAGTCAGGA
WT           TCTCTCGCCCATGTACCATC-TCCATGG---------------TTACTGAGTCATTATGTGATC-GTCAGGA
                 TCGCCCATGTACCATC-TCCA                      TGAGTCATTATGTGATC-GTC
```

CRISPR-*OsDof24*:

```
+1 T         ACCATTTGCTTGCCCCCGCTCCTCGG-----------------CCGTCGACCTGCGCCGTCCTCAAGGG
+1 A         ACCATTTGCTTGCCCCCGCTCCTCGG-----------------CCGTCGACCTGCGCCGTCCACAAGGG
WT           ACCATTTGCTTGCCCCCGCTCCTCGG-----------------CCGTCGACCTGCGCCGTCC-CAAGGG
                ATTTGCTTGCCCCCGCTCCT                      GTCGACCTGCGCCGTCC-CAA
```

CRISPR-*OsDof25*:

```
-117         CAGAAGCCACAACCAAGAAAGC---------------------------------------------GTCGGG
WT           CAGAAGCCACAACCAAGAAAGCCCCGG----------------- CACGAGCCCGTTCGAGTGGCCGTCGGG
                 AGCCACAACCAAGAAAGCCC                         AGCCCGTTCGAGTGGCCGTC
```

CRISPR-*OsDof26*:

```
+1 T, -720   CCGGTGTCAGCCGGATGCCAAGGTTGG------TGTATGT----------------------------------- GGCAG
WT           CCGGTG-CAGCCGGATGCCAAGGTTGG------TGTATGTCTCT------TTGCAGGATGTTCAGCAGCATAGTGGCAG
                GTG-CAGCCGGATGCCAAGGT                          CAGGATGTTCAGCAGCATAG
```

CRISPR-*OsDof27*:

```
-212         TCCGACATGCGCAGGGGTTC------------------------------------CCTAGG---------GCGGCGGCGCCGAGG
-399         TCCGACATGCGCAGGGGTTCA---------------------------------------------------------- GCCGAGG
WT           TCCGACATGCGCAGGGGTTCACCGTGG--------CCCTCTCTTACCTGACCCCGCCTAGG---------GCGGCGGCGCCGAGG
                 ACATGCGCAGGGGTTCACCG                TCTCTTACCTGACCCCGCCT
```

CRISPR-*OsDof28*:

```
-425         TCCAATCTTCCTAGACCCTCCA-------------------------------------------CGGCGGC
-426         TCCAATCTTCCTAGACCCTCC--------------------------------------------CGGCGGC
WT           TCCAATCTTCCTAGACCCTCCAAATTGGA--------------CAGCCACTCCAGTGTCTCATCGGCGGC
                  TCTTCCTAGACCCTCCAAAT                     CCACTCCAGTGTCTCATCGG
```

CRISPR-*OsDof29*:

```
-4           CGGCGGCGTTCTCTCACC----CCACGG------------------CTGGTGCAACGCGTTGGGTTGCCAAGG
-1 T         CGGCGGCGTTCTCTCACCGG-TCCACGG------------------CTGGTGCAACGCGTTGGGTTGCCAAGG
WT           CGGCGGCGTTCTCTCACCGGTTCCACGG------------------CTGGTGCAACGCGTTGGGTTGCCAAGG
                   GCGTTCTCTCACCGGTTCCA                        TGCAACGCGTTGGGTTGCCA
```

CRISPR-*OsDof30*:

```
-210         CTCCCAGA-----------------------------------------------------------------GCTGTGGC
-5, +1 A     CTCCCAGA------GCCCAGCAGCAGCAGG--------------------TCGTACTCCCCGTTCCCAGCGGCTGTGGC
-1 T, -1 G   CTCCCAGA--TGCCGCCCAGCAGCAGCAGG-------------------- TCGTACTCCCCGTTCCCAGC--CTGTGGC
+1 T, -1 G   CTCCCAGATTTGCCGCCCAGCAGCAGCAGG-------------------- TCGTACTCCCCGTTCCCAGC--CTGTGGC
WT           CTCCCAGA-TTGCCGCCCAGCAGCAGCAGG-------------------- TCGTACTCCCCGTTCCCAGC-GCTGTGGC
                  AGA-TTGCCGCCCAGCAGCAG                            ACTCCCCGTTCCCAGC-GCTG
```

Figure S3 CRISPR/Cas9-induced mutations in the *Dof* genes in T_0 generation plants.

The targeted sequence is highlighted in red and the mutations are marked in green color.

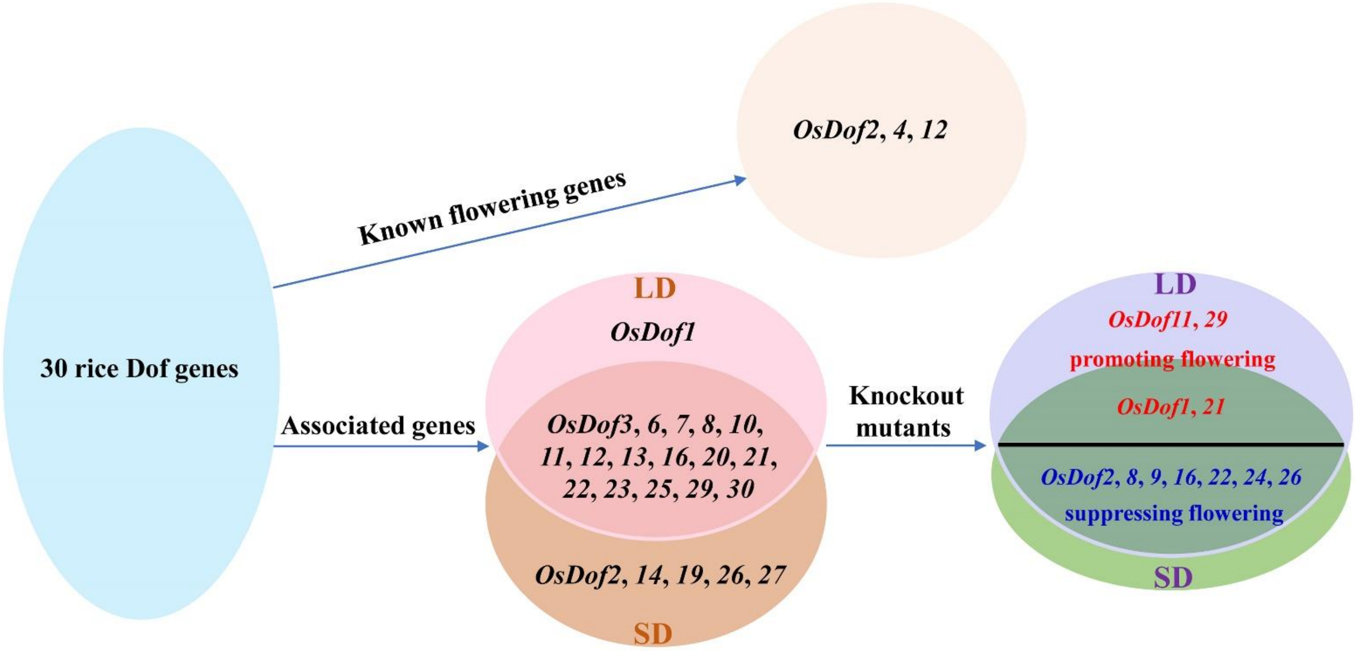

Figure S4 A diagram showing the 30 *Dof* genes and findings from association tests and CRISPR/Cas9 experiments.
